# Supplementary material for: The role of internal transcribed spacer 2 secondary structures in classifying mycoparasitic Ampelomyces
Source: PLoS One. 2021 Jun 30;16(6):e0253772. doi: 10.1371/journal.pone.0253772 (PMC8244850; doi:10.1371/journal.pone.0253772)

## Slide 1
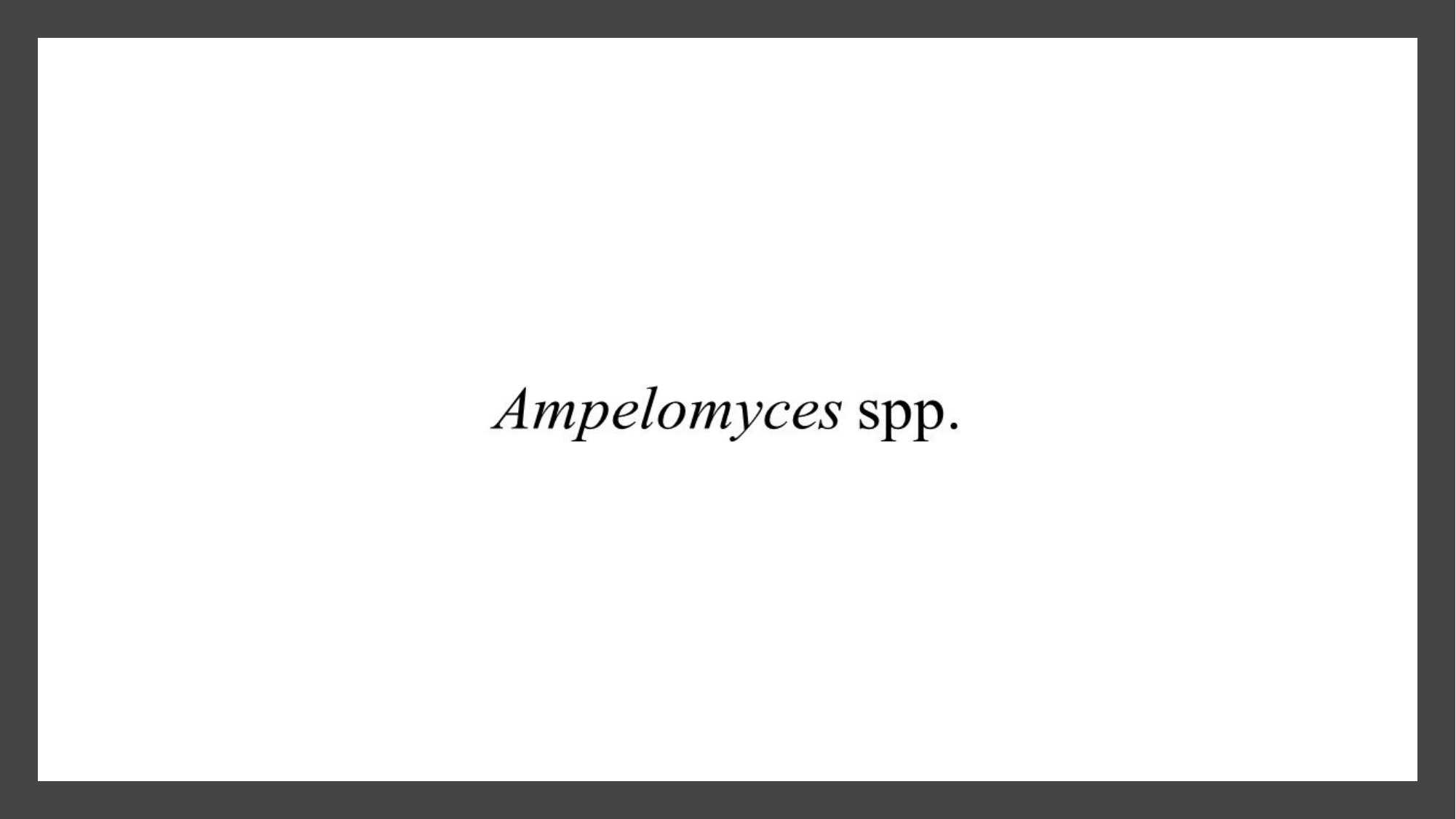

## Slide 2
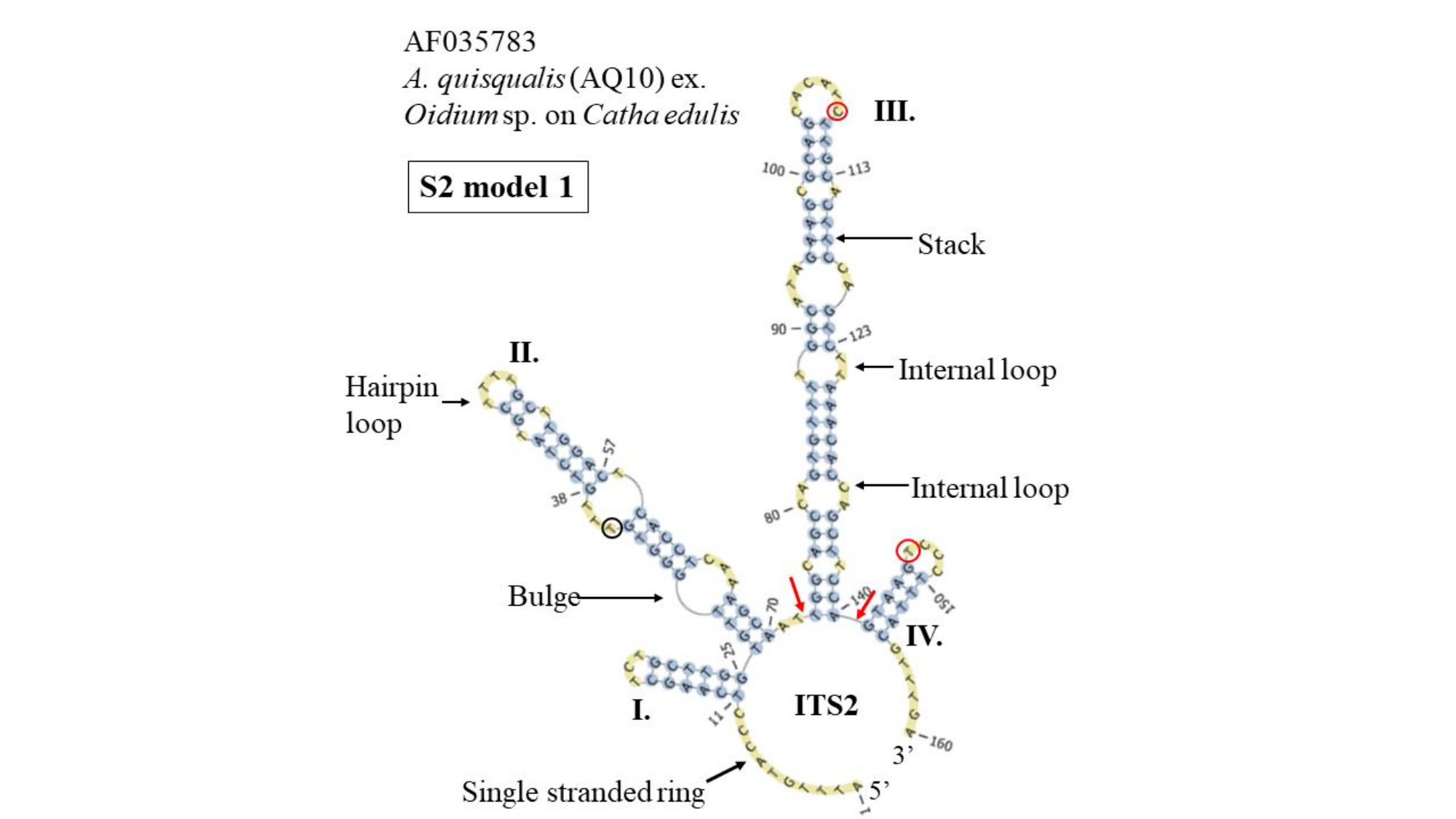

## Slide 3
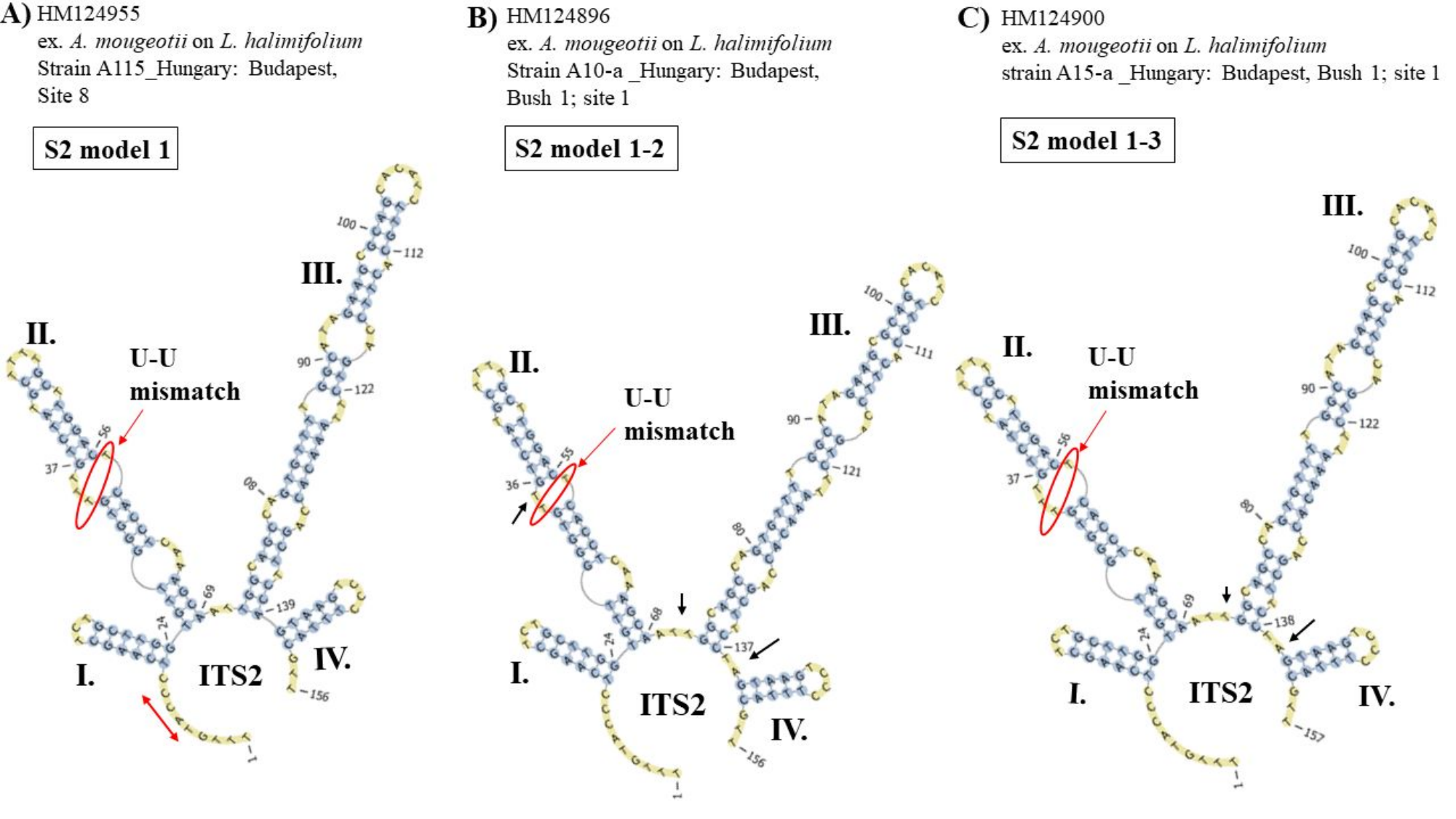

## Slide 4
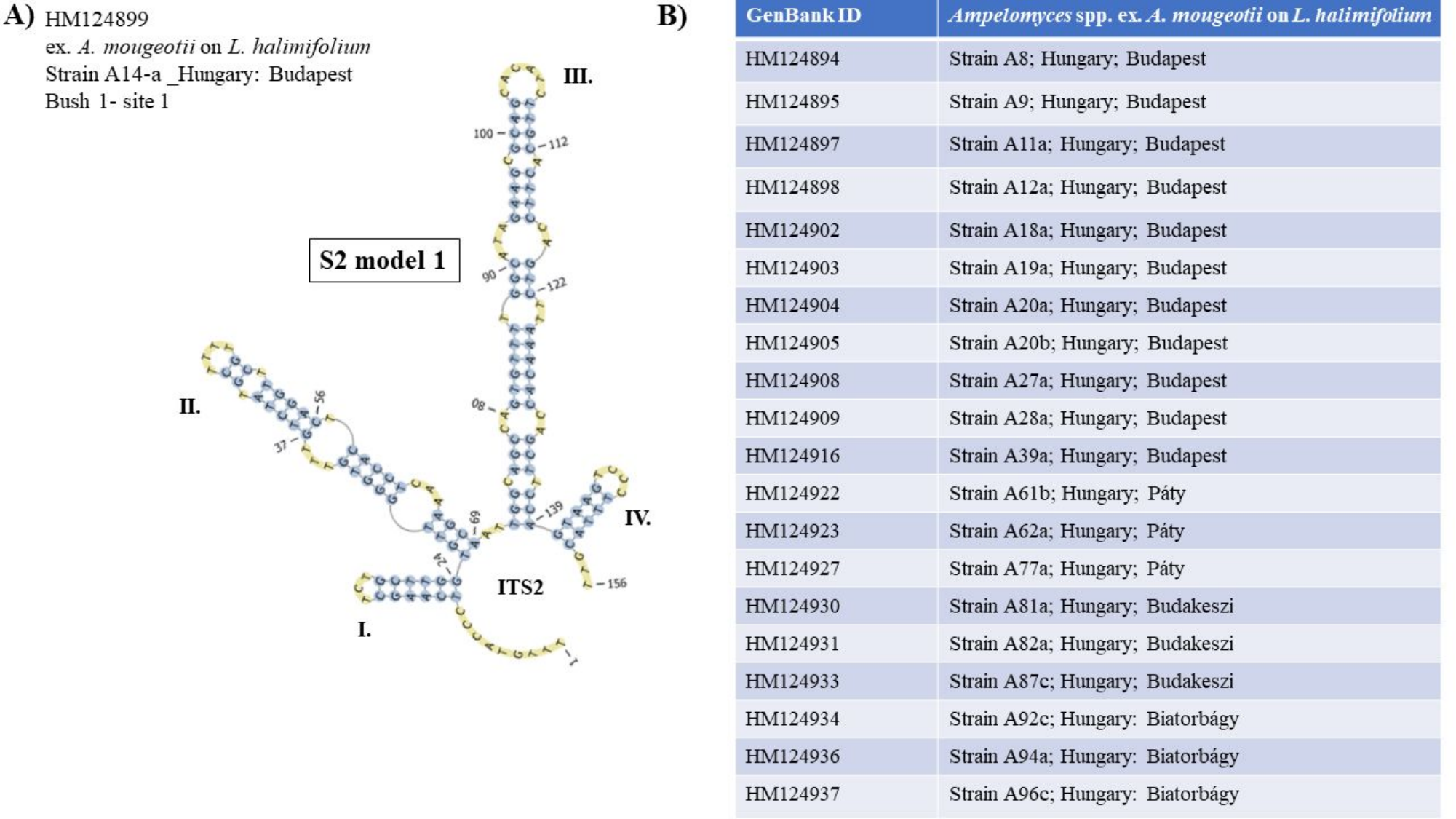

## Slide 5
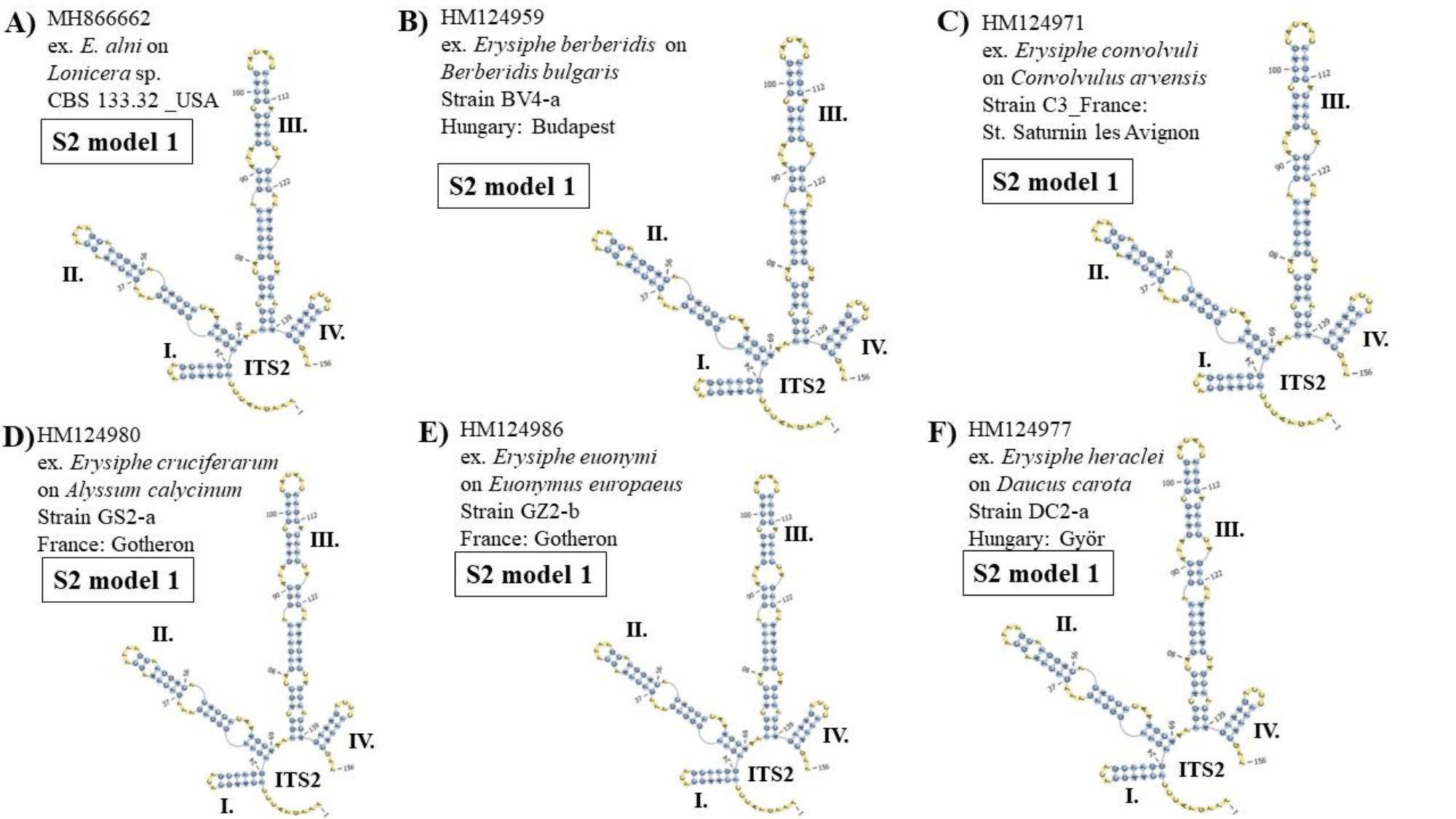

## Slide 6
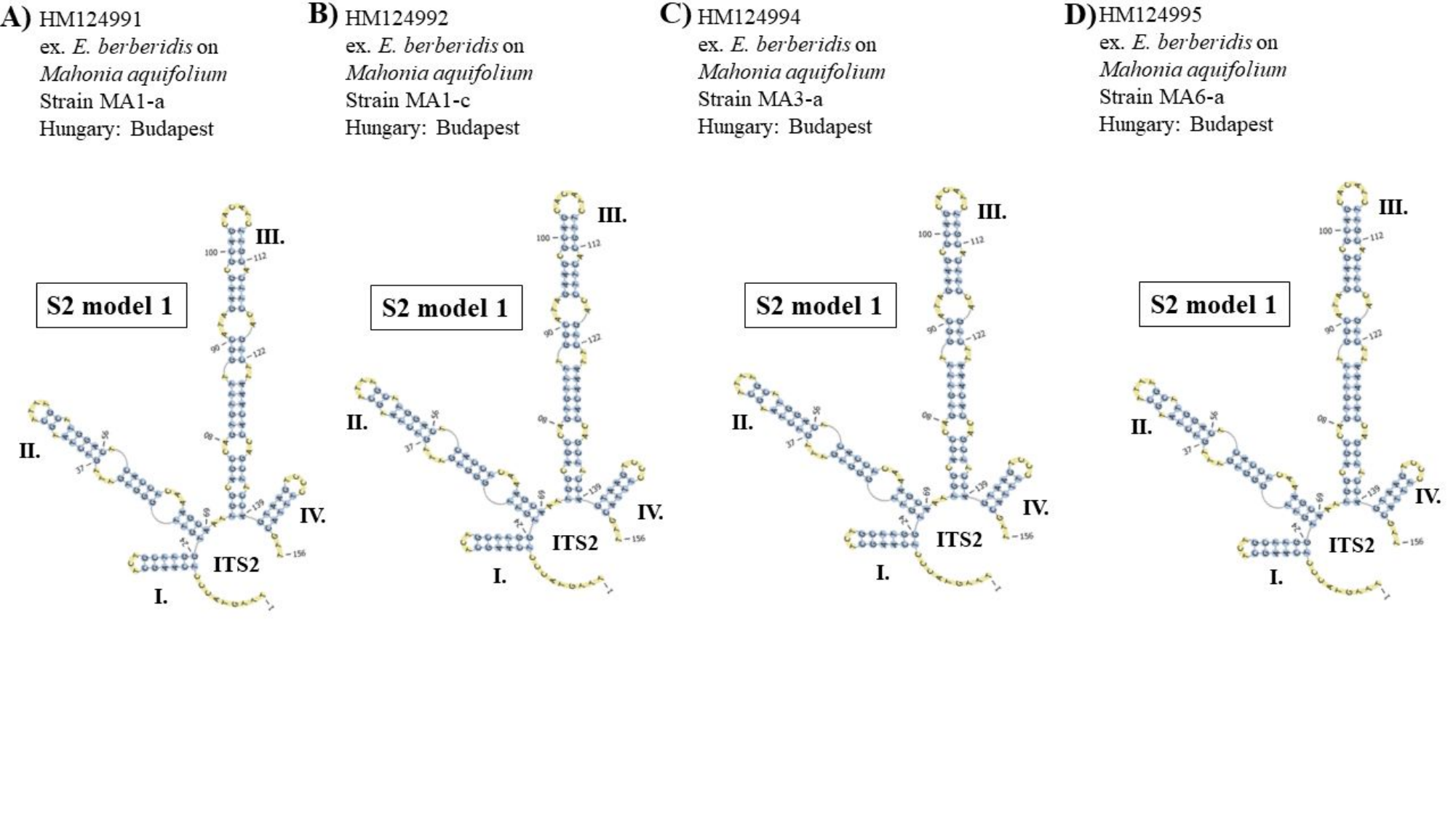

## Slide 7
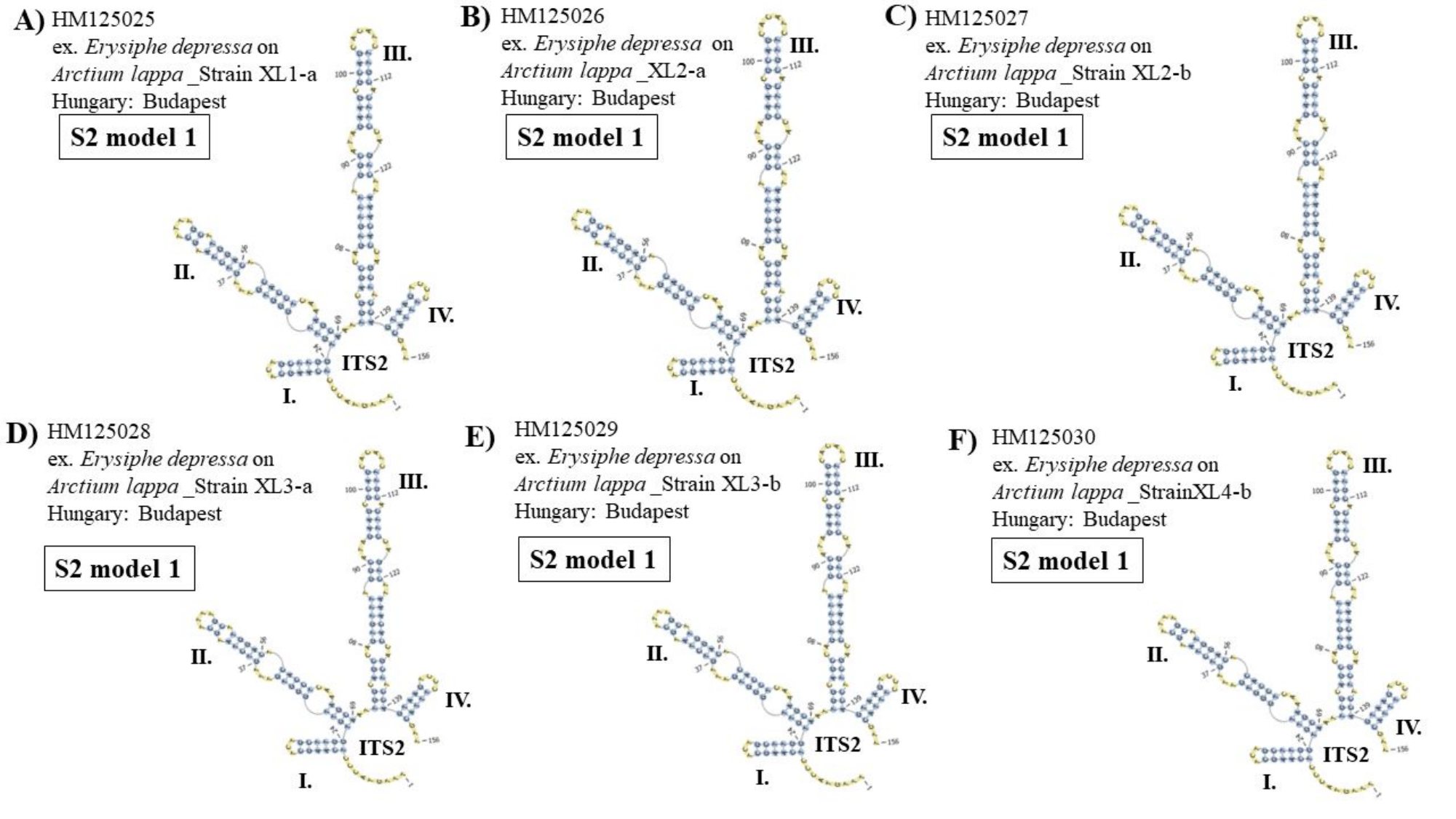

## Slide 8
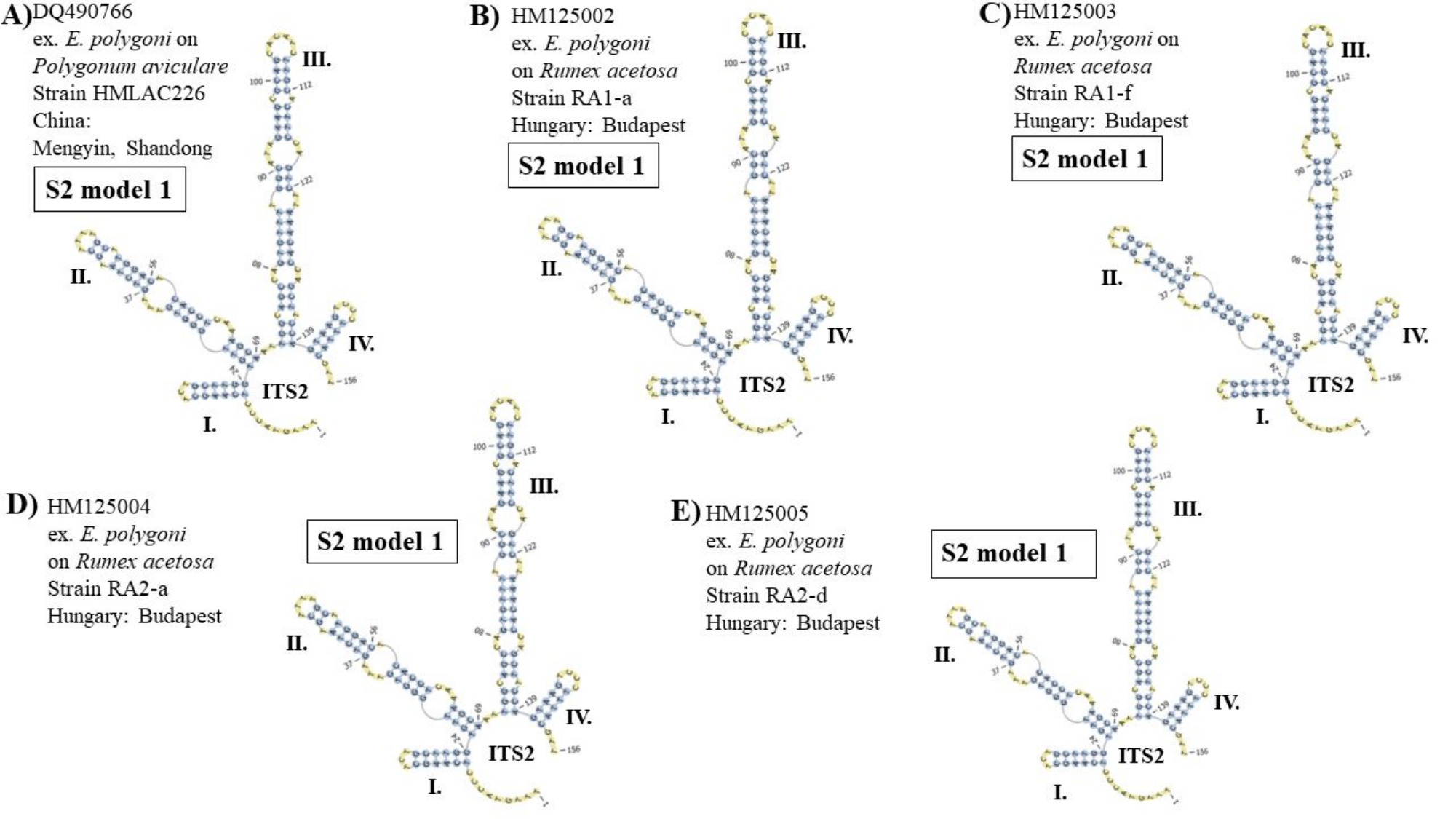

## Slide 9
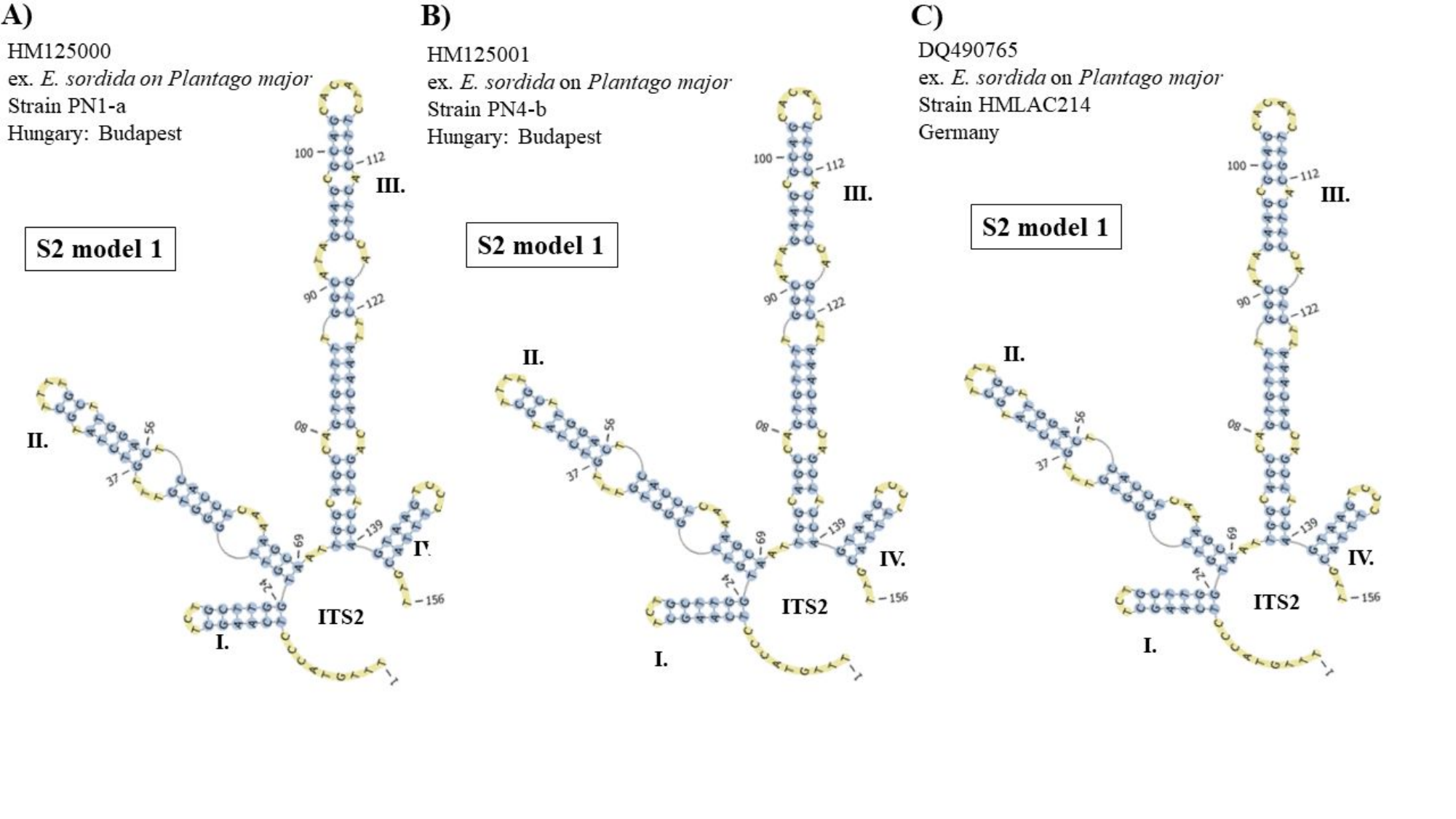

## Slide 10
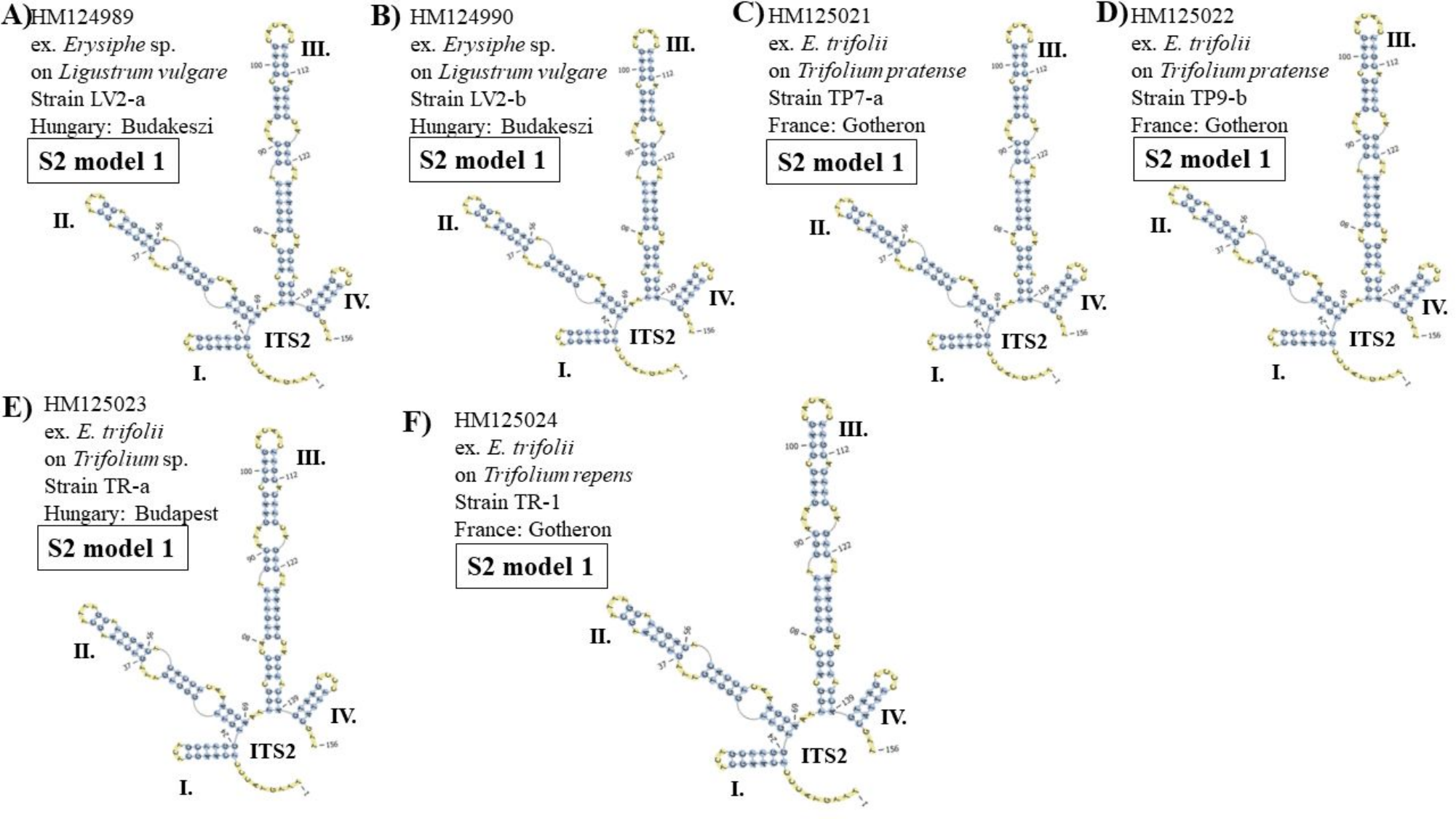

## Slide 11
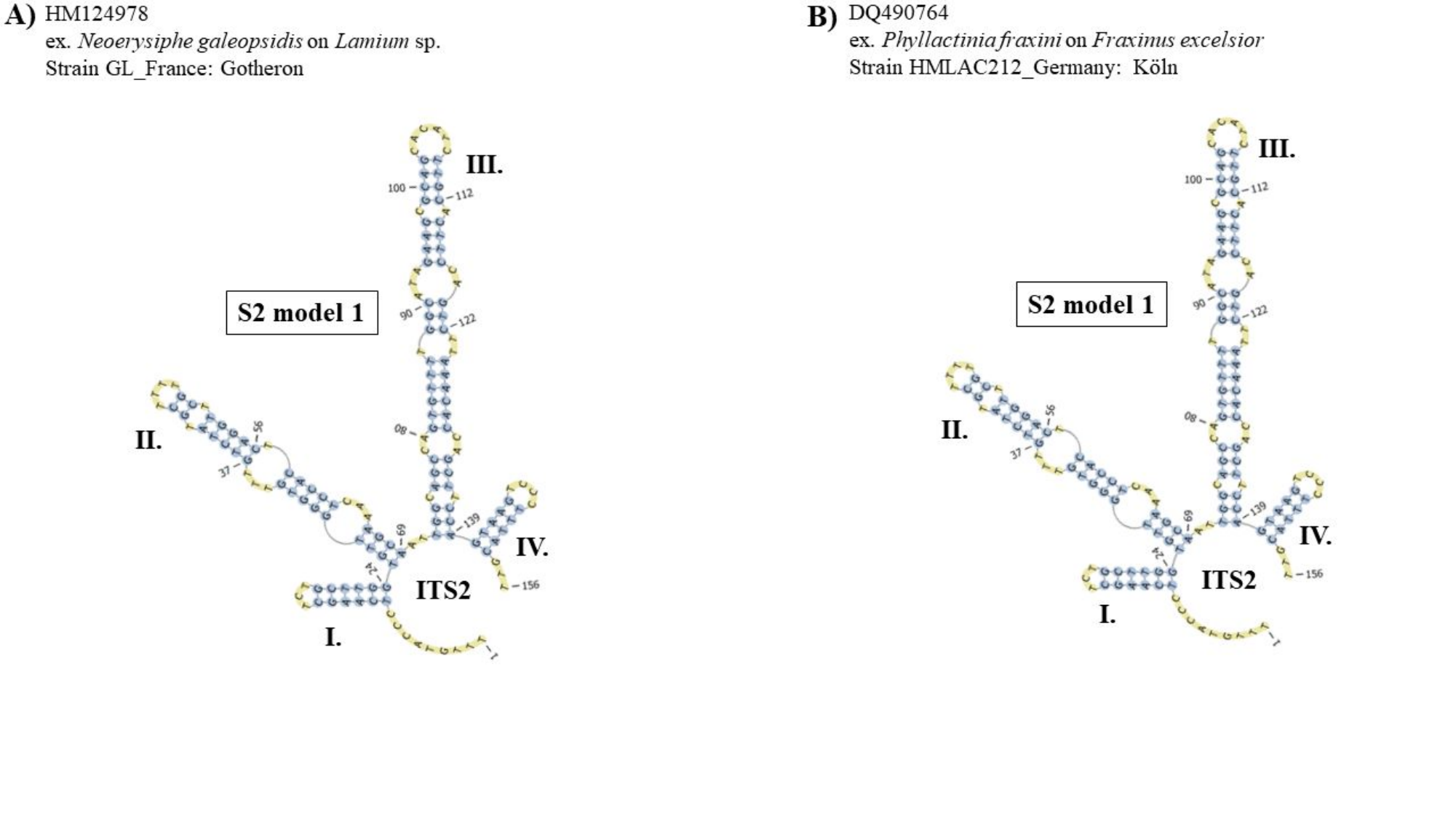

## Slide 12
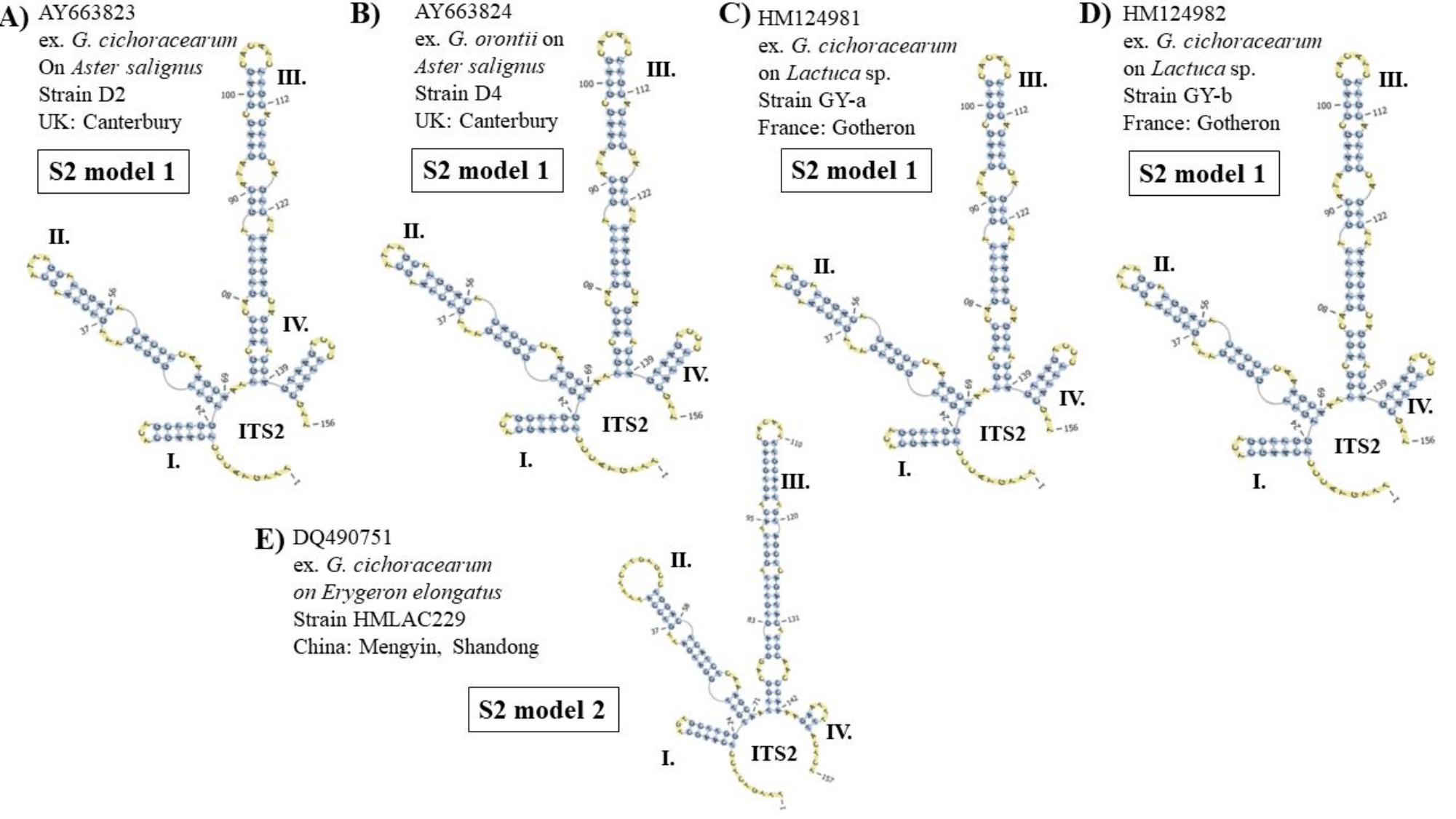

## Slide 13
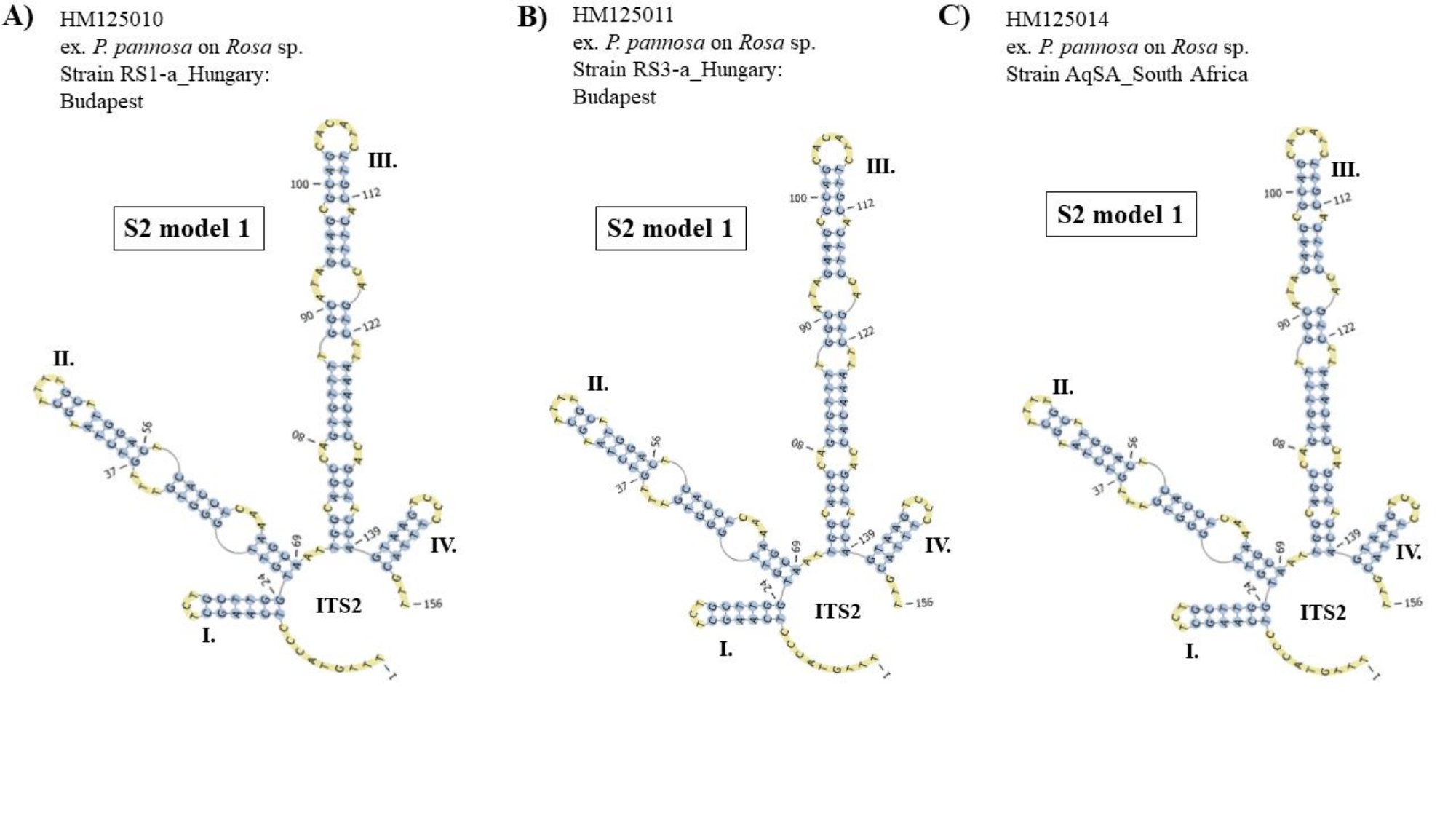

## Slide 14
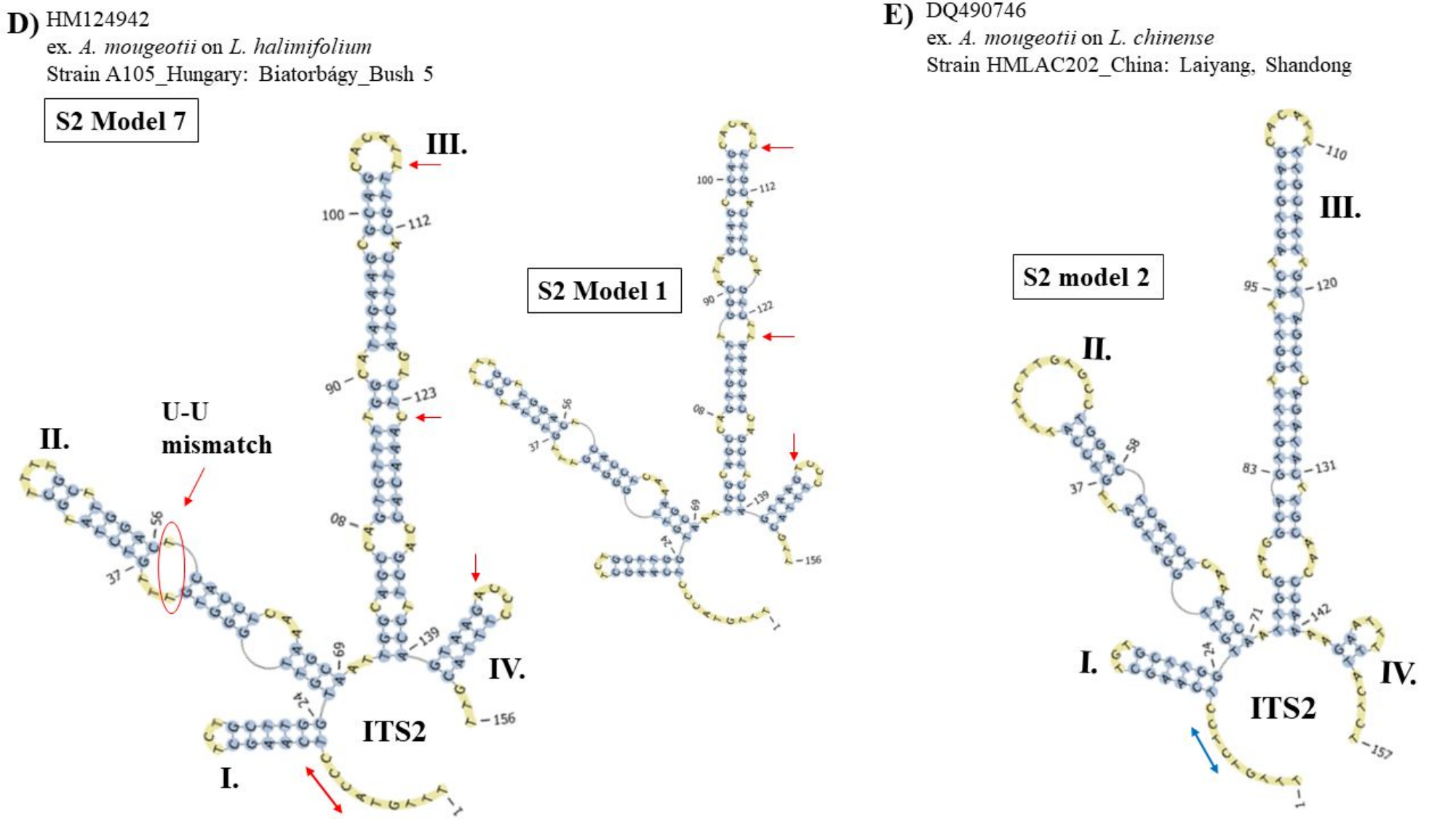

## Slide 15
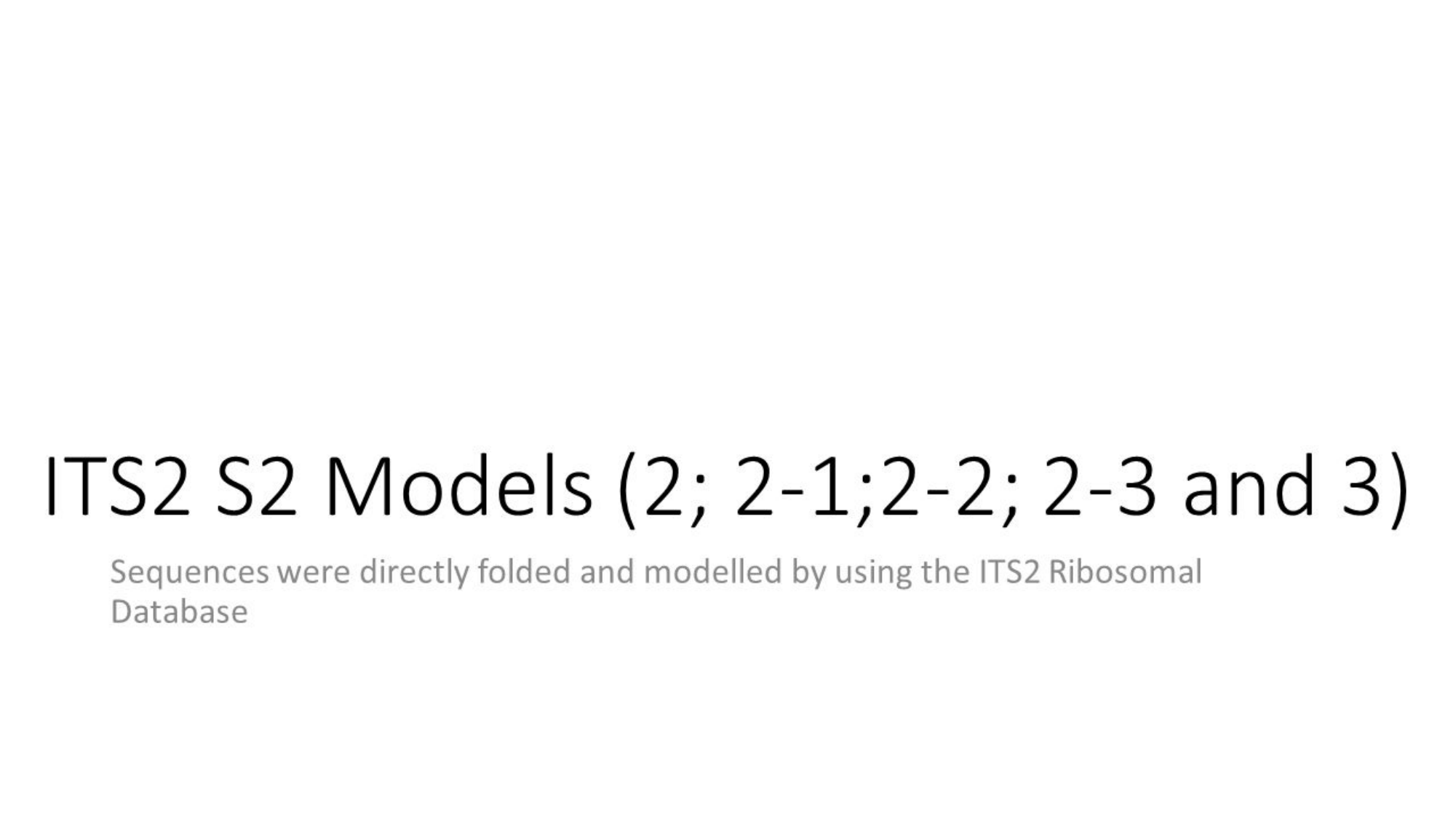

## Slide 16
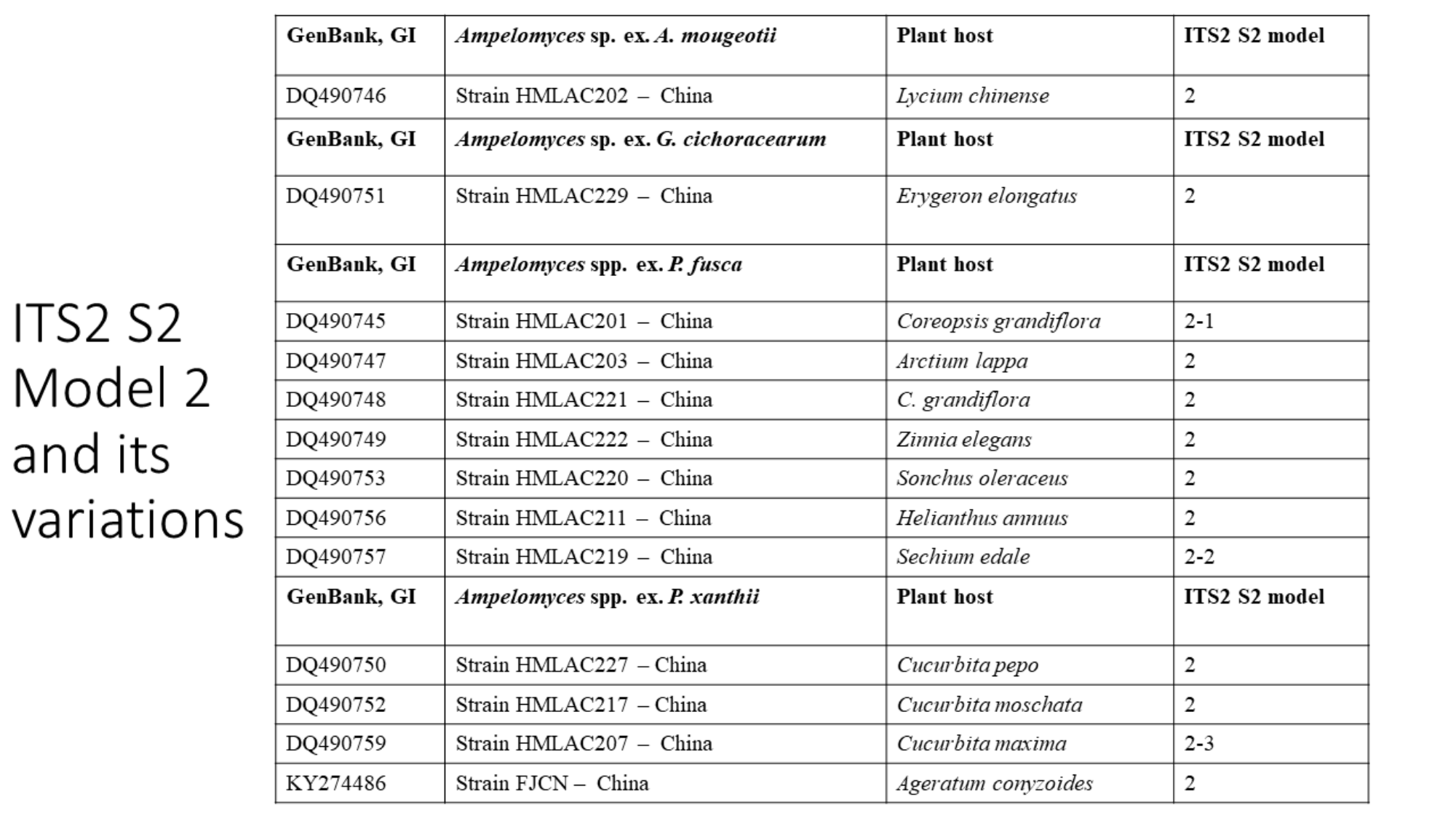

## Slide 17
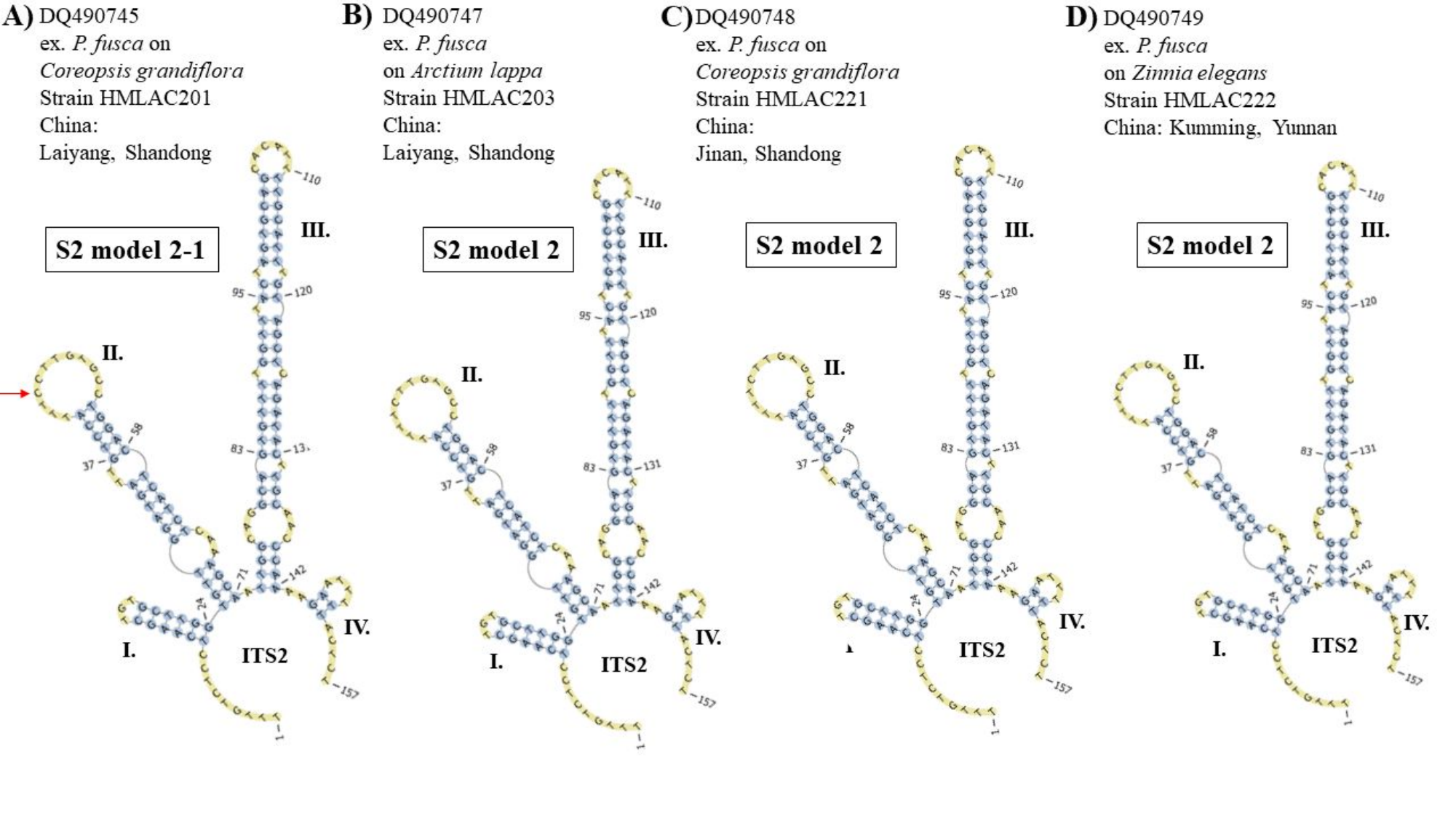

## Slide 18
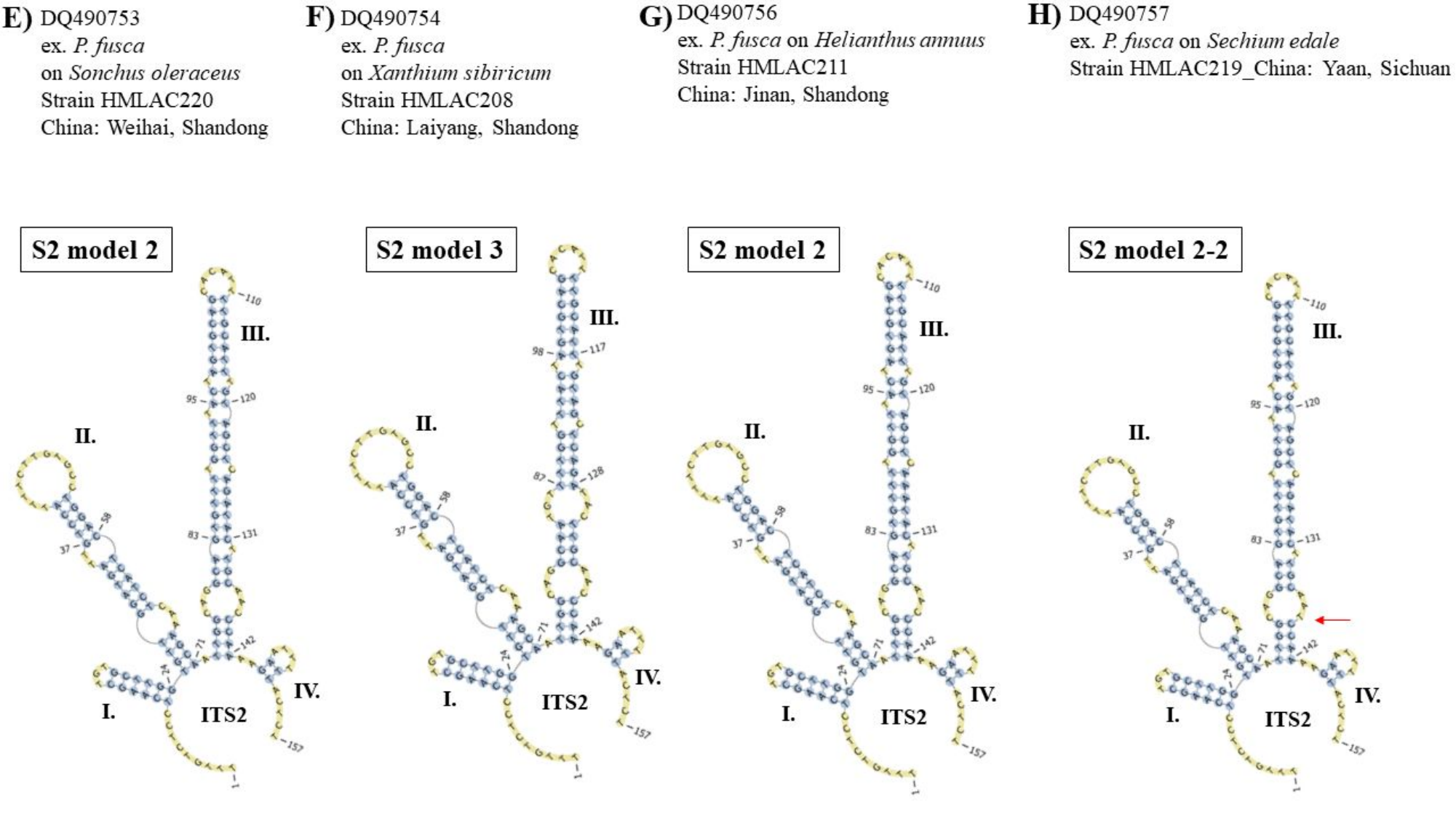

## Slide 19
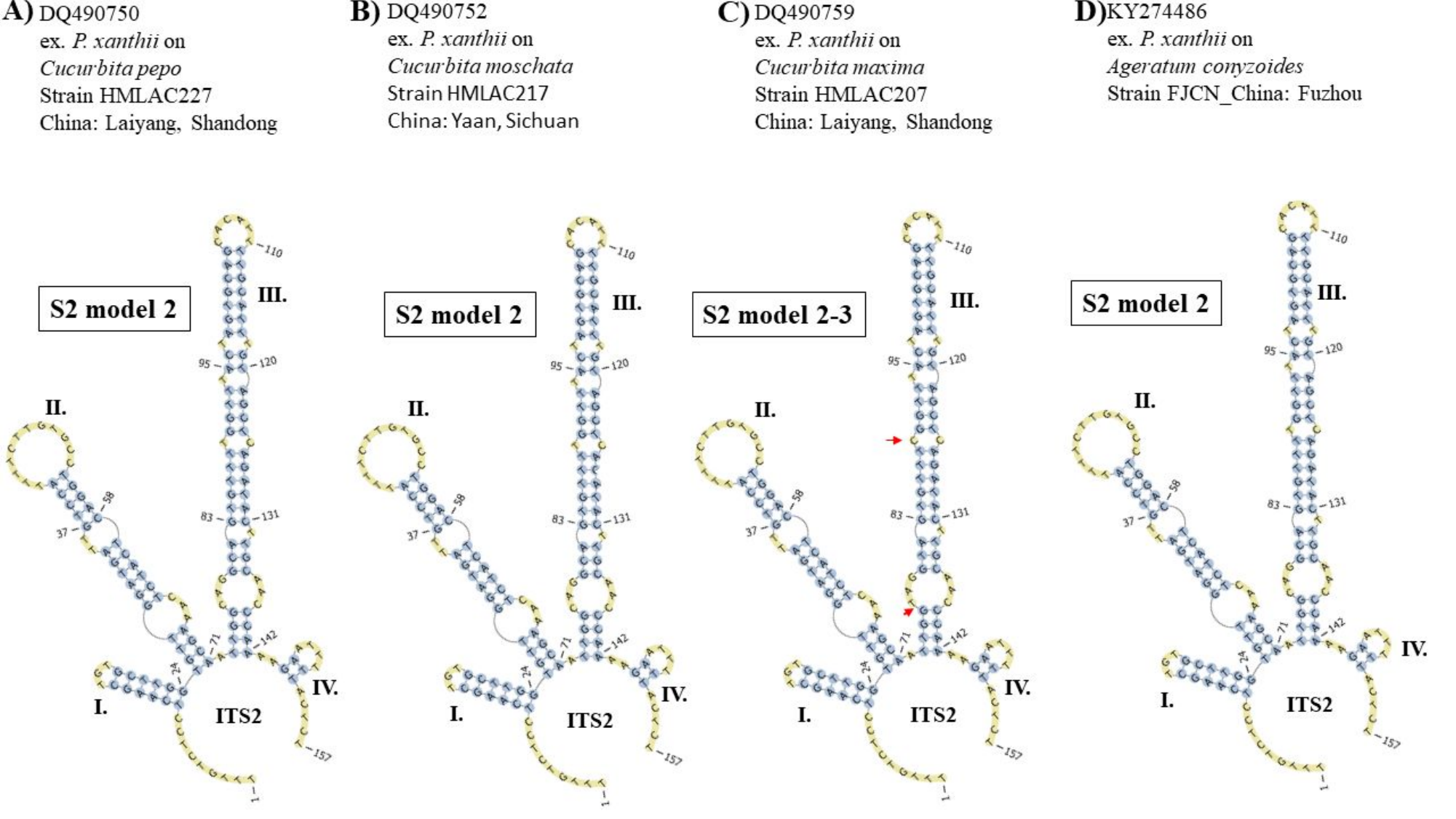

## Slide 20
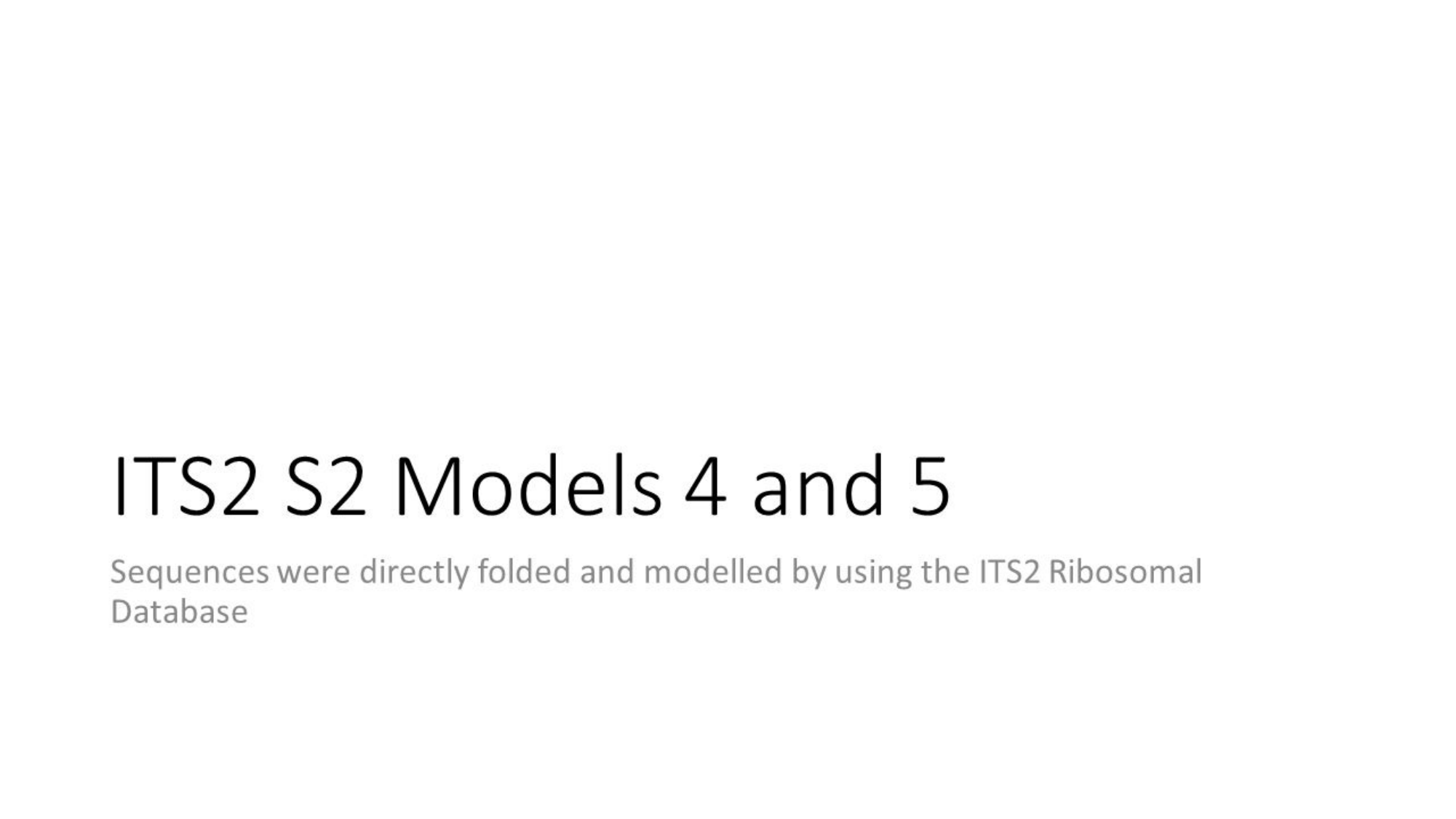

## Slide 21
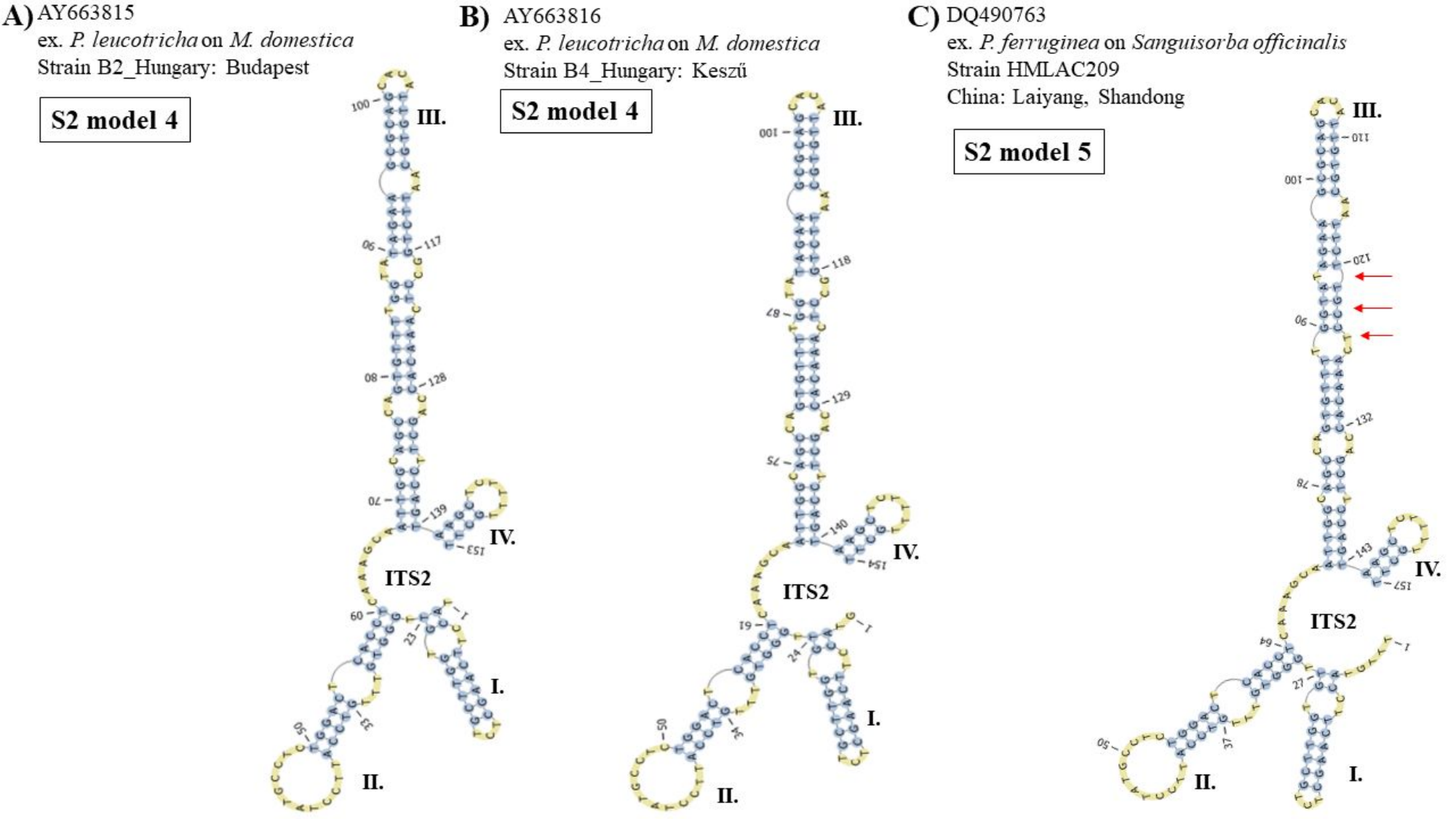

## Slide 22
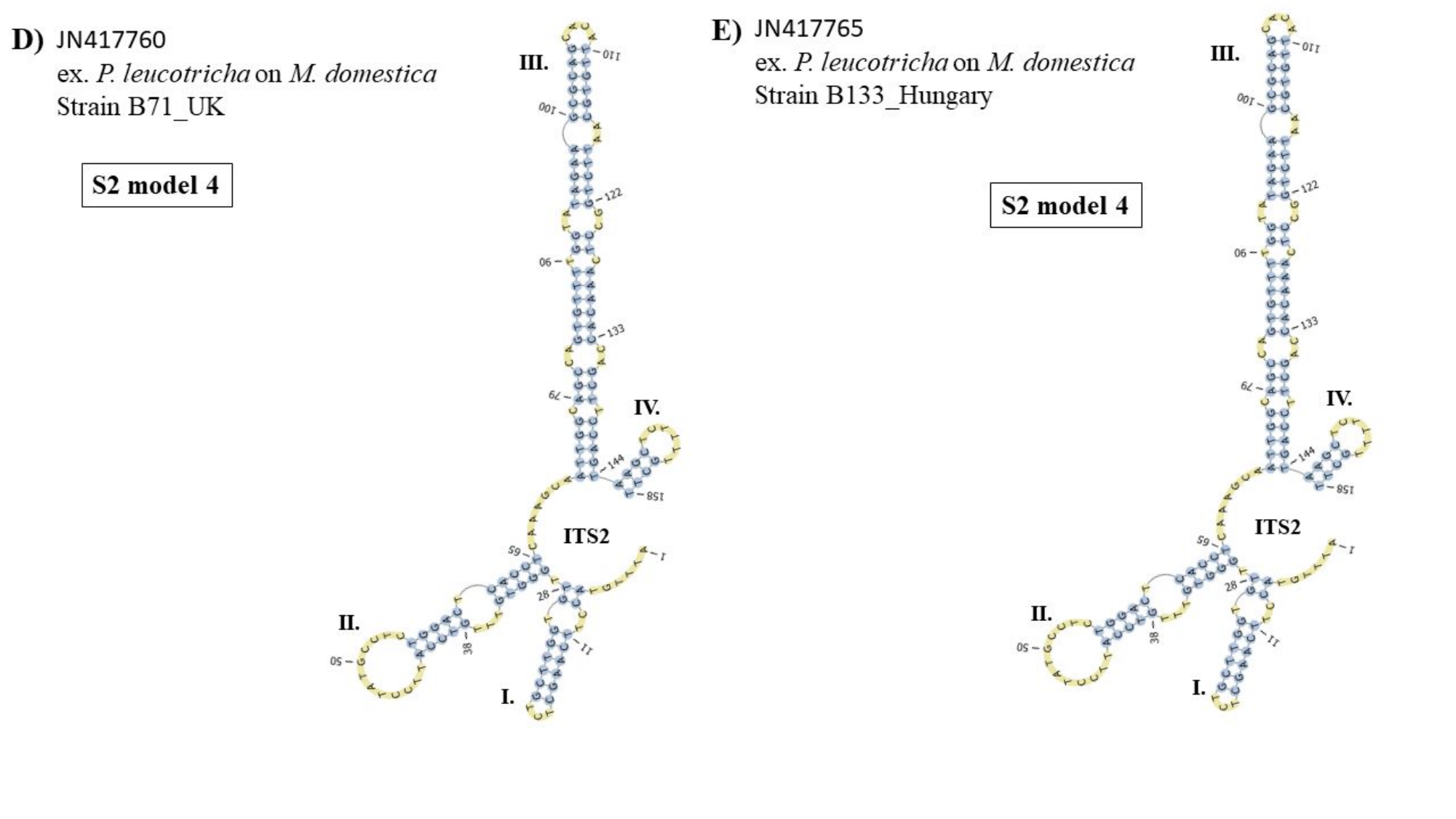

## Slide 23
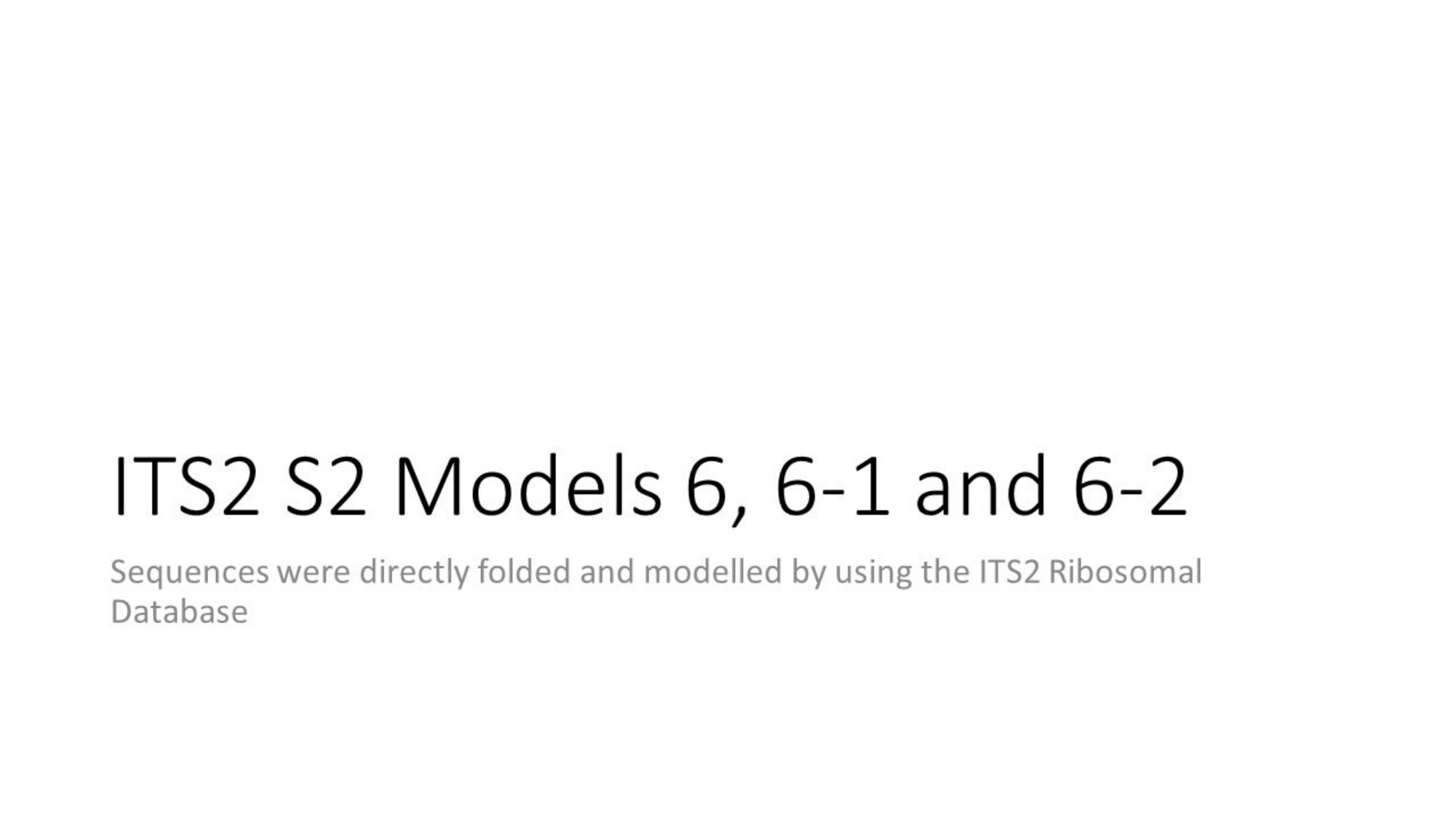

## Slide 24
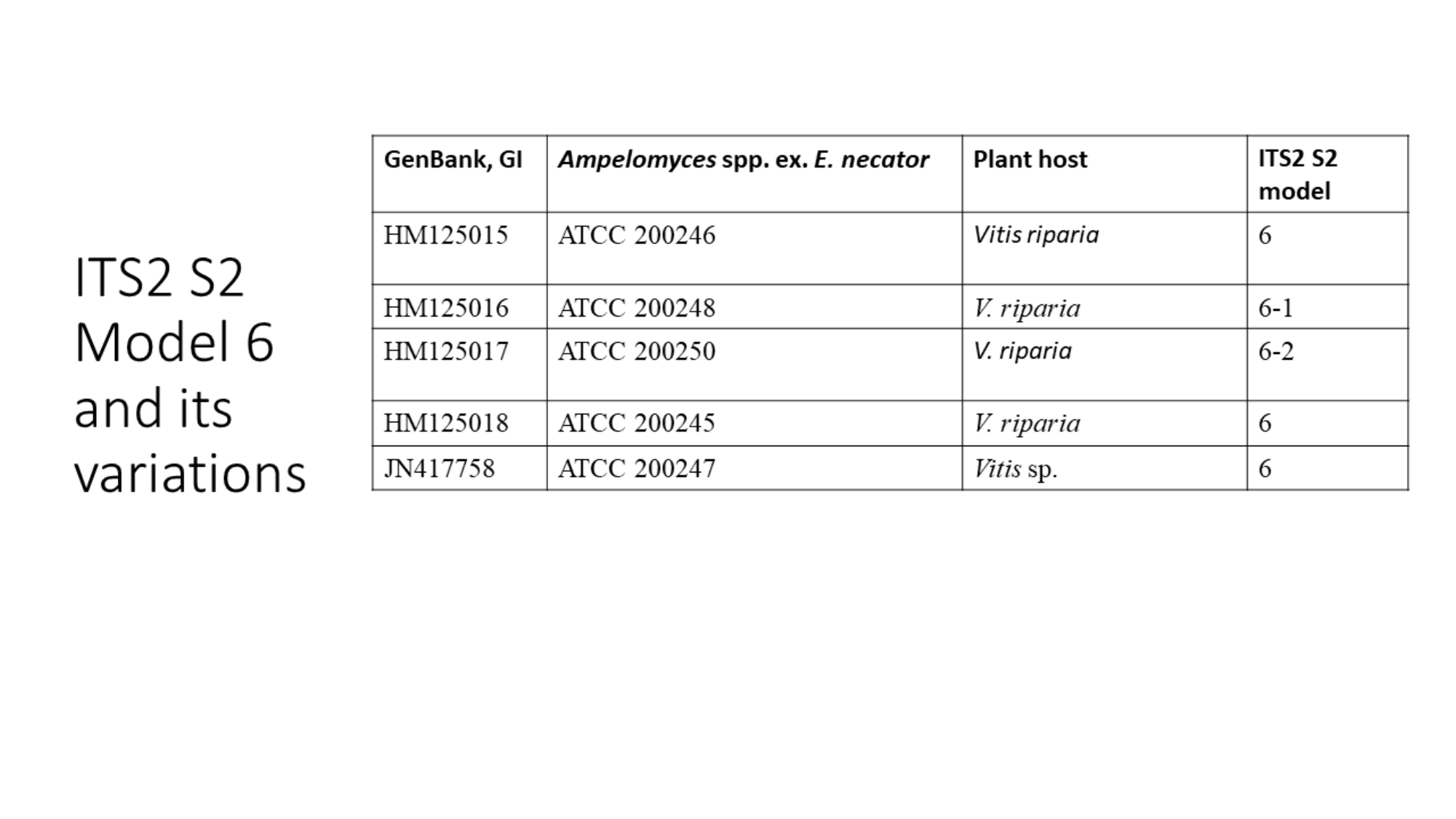

## Slide 25
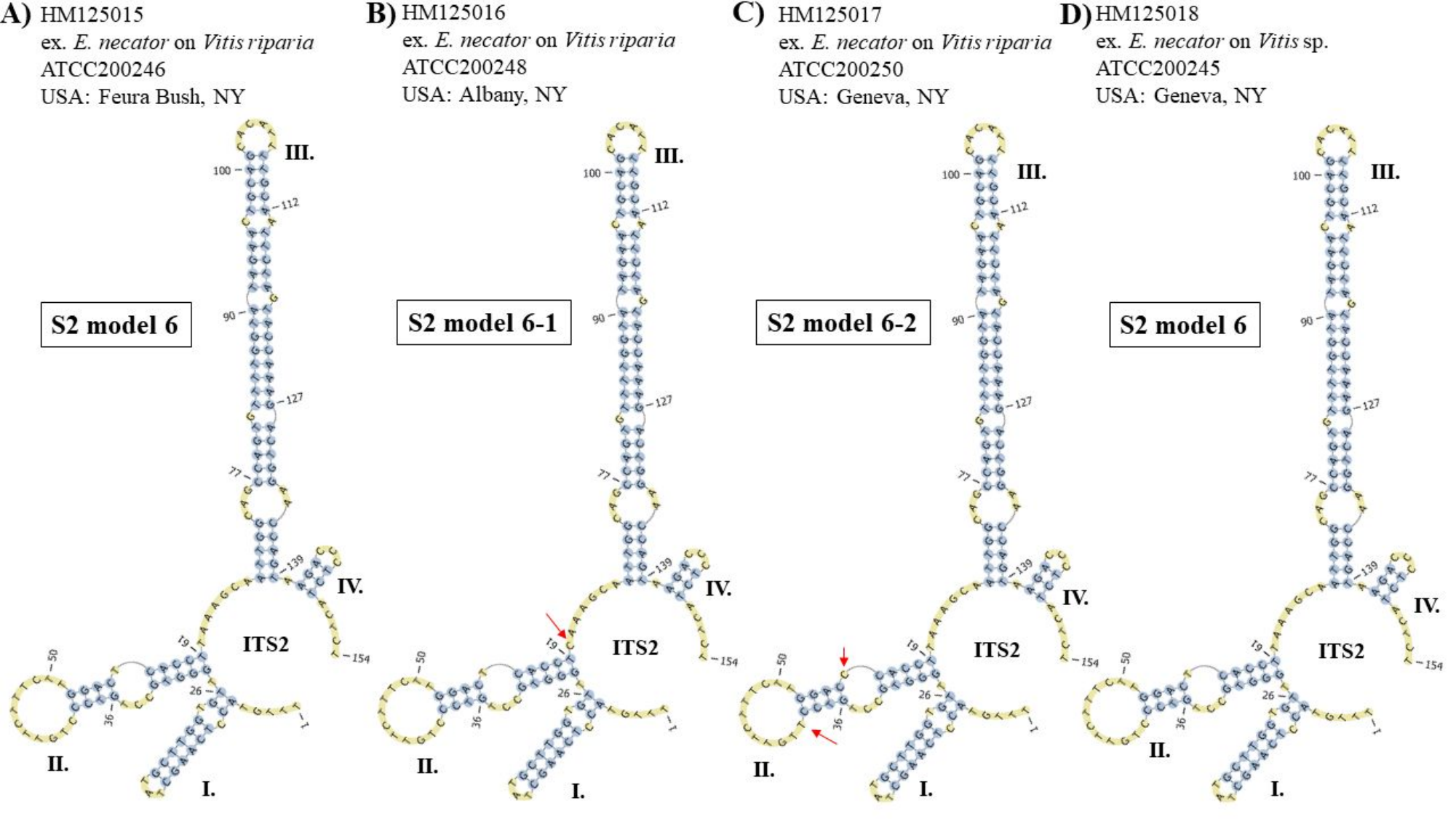

## Slide 26
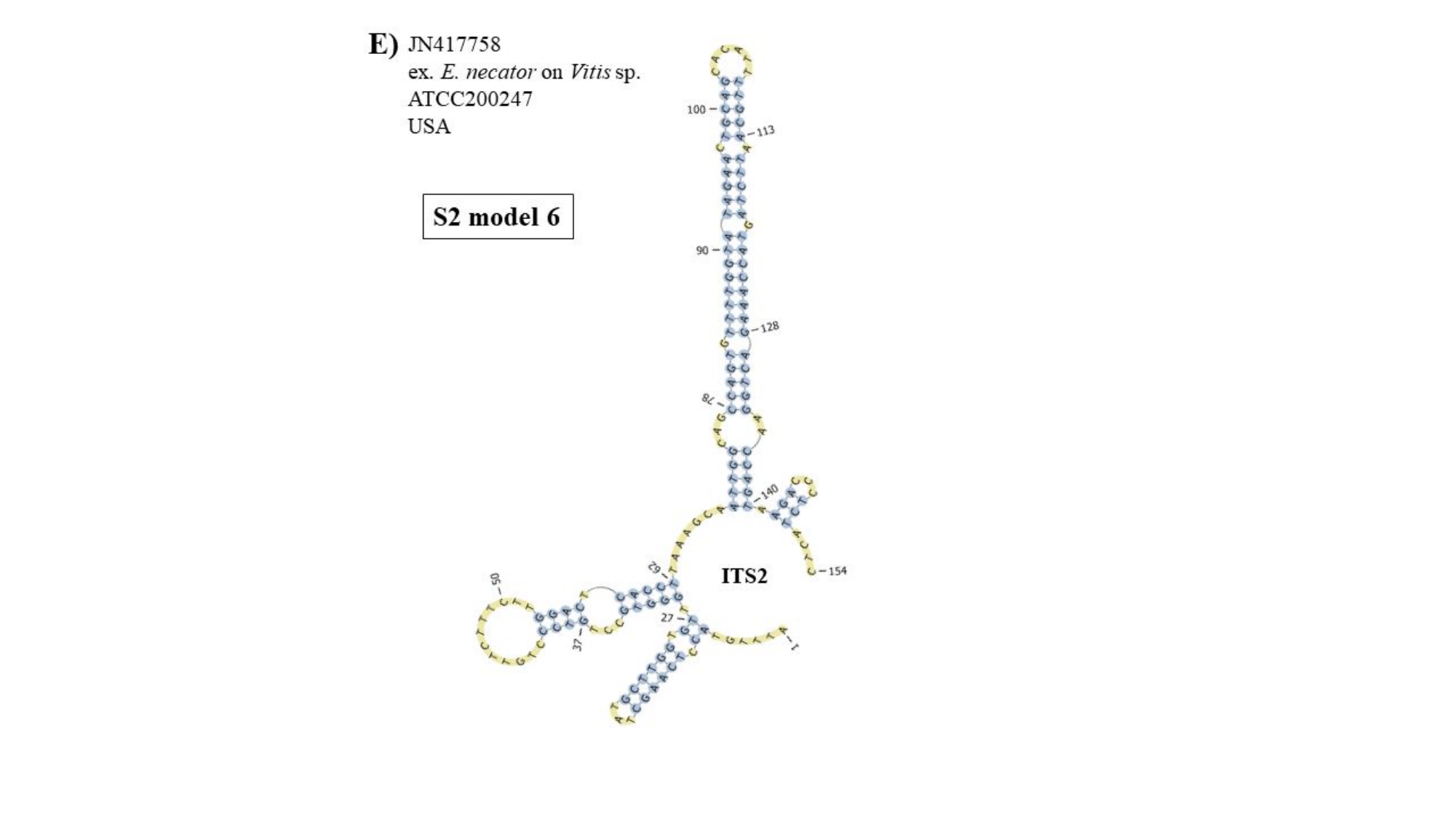

## Slide 27
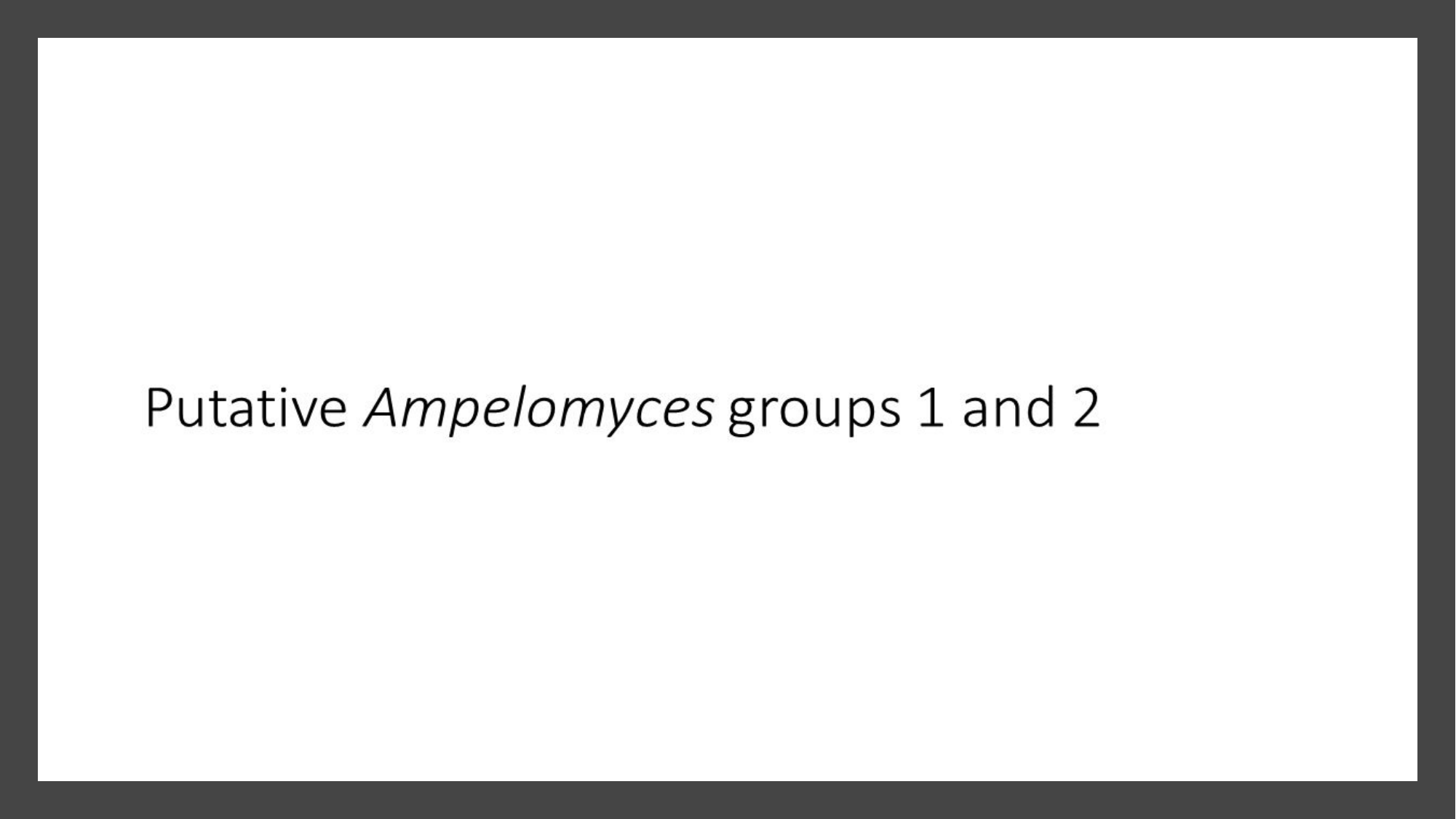

## Slide 28
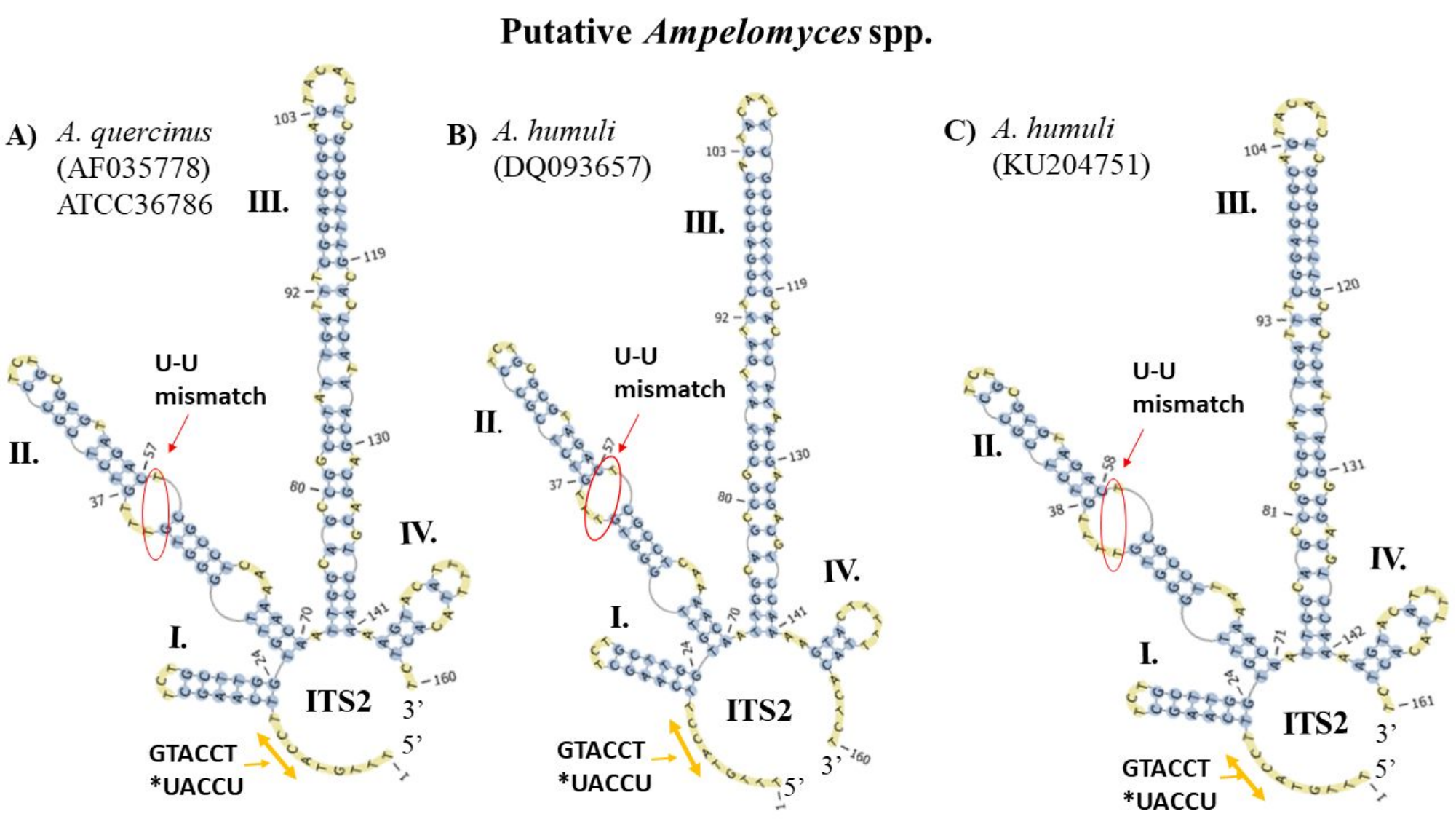

## Slide 29
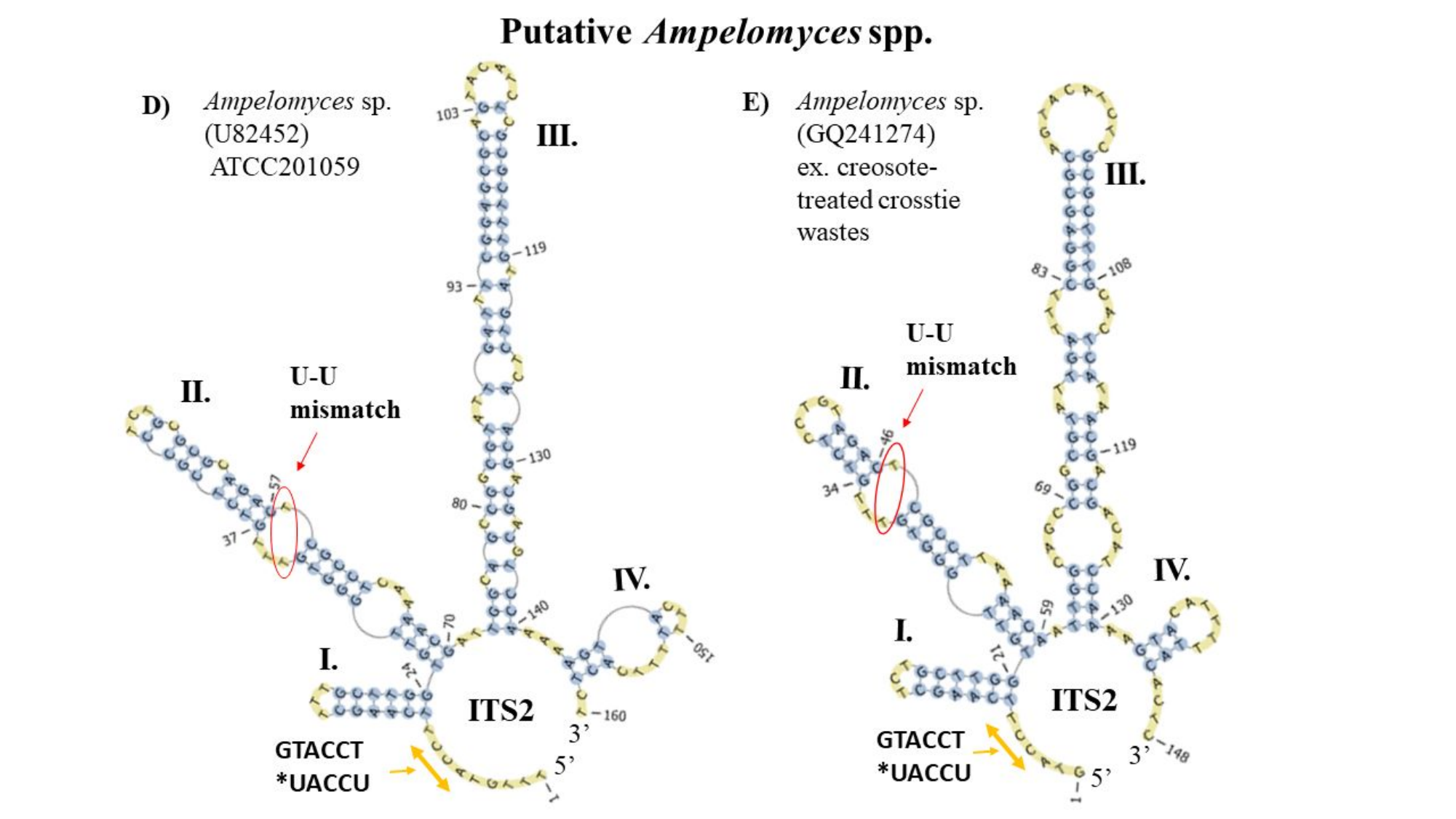

Supplement: S2 File — (ZIP) [file pone.0253772.s002.zip › Supplemental File 2 Prahl et al 2021 PLOSONE.pptx]
