# Supplementary material for: The role of internal transcribed spacer 2 secondary structures in classifying mycoparasitic Ampelomyces
Source: PLoS One. 2021 Jun 30;16(6):e0253772. doi: 10.1371/journal.pone.0253772 (PMC8244850; doi:10.1371/journal.pone.0253772)
Supplement: Attachment — Submitted filename: Response to Reviewers.pdf [file pone.0253772.s005.pdf]

Responses to the Review Comments

03 June 2021

Dear Editor,

We are really thankful you and to all the reviewers for their valuable comments; and we have edited the final manuscript addressing all concerns raised by the reviewers.

There are no further review comments/suggestions other than the review comment (6) from Reviewer 2. Our responses are noted below:

1. Keywords are included below the abstract (Lines 46 and 47)
2. References citations are substituted in the text by square brackets (eg. Line 55).
3. Paragraph (Lines 73—93) are re-written for clarity (Please see in the comment Line 73): …genus or family. For example, if some taxa have low ITS interspecific variability other molecular markers need to be used to precisely report genetic diversity [9]. Conversely, intragenomic variation of the ITS can result in overestimate the intraspecific variability in some fungi [9–12]. Thus, relying in the use of only one molecular marker in some fungi can result in poor species discrimination resolution and other molecular barcodes may be required as the accuracy of the identification can be compromised. Therefore, it is crucial to have an optimum barcode gap.
4. The em dashes (—) are correctly added across all the document, i.e., Line 94.
5. Data extraction was substituted by Sequence acquisition (Line 135).
6. *Ampelomyces quercinus* was written in full (Line 149).
7. Paragraph (Lines 547 and 548) are edited for clarification: …infecting several PM species representing seven genera, specifically from …
8. Paragraph (Lines 557) are edited for clarification: …. *Golovinomyces cichoracearum on Aster salignus* and on *Lactuca* sp.
9. The powdery mildew species were written in alphabetic order. *Phyllactinia fraxini* was placed before *Podosphaera pannosa* (Line 558 and 559).
10. Fonts were corrected and checked across all the document (Lines 1015—1016).
11. All references are formatted according to guidelines of PLoS One (Lines 1071—1251).
12. Figures were also formatted i.e., (Fig 1).
13. Supporting information was moved at the end of the References (Lines 1254—1258).
14. File S3 contains the curated *Ampelomyces* ITS nucleotide sequences (Line 1258).

We think the manuscript is ready for publication in PLoS One journal.

Best wishes,

Rosa E. Prahl

U1114537@umail.usq.edu.au

On behalf of all the authors
